# Silver Nanoparticles Modified by Carbosilane Dendrons and PEG as Delivery Vectors of Small Interfering RNA

**DOI:** 10.3390/ijms24010840

**Published:** 2023-01-03

**Authors:** Viktar Abashkin, Elżbieta Pędziwiatr-Werbicka, Katarzyna Horodecka, Victoriya Zhogla, Egor Ulashchik, Vadim Shmanai, Dzmitry Shcharbin, Maria Bryszewska

**Affiliations:** 1 Institute of Biophysics and Cell Engineering of NASB, 27 Akademicheskaya St., 220072 Minsk, Belarus; 2 Department of General Biophysics, Faculty of Biology and Environmental Protection, University of Lodz, 141/143 Pomorska St., 90-236 Lodz, Poland; 3 Institute of Physical Organic Chemistry of NASB, 13 Surganova St., 220072 Minsk, Belarus

**Keywords:** silver nanoparticles, carbosilane dendrons, PEGylation, siRNA, gene therapy

## Abstract

The fact that cancer is one of the leading causes of death requires researchers to create new systems of effective treatment for malignant tumors. One promising area is genetic therapy that uses small interfering RNA (siRNA). These molecules are capable of blocking mutant proteins in cells, but require specific systems that will deliver RNA to target cells and successfully release them into the cytoplasm. Dendronized and PEGylated silver nanoparticles as potential vectors for proapoptotic siRNA (siMCL-1) were used here. Using the methods of one-dimensional gel electrophoresis, the zeta potential, dynamic light scattering, and circular dichroism, stable siRNA and AgNP complexes were obtained. Data gathered using multicolor flow cytometry showed that AgNPs are able to deliver (up to 90%) siRNAs efficiently to some types of tumor cells, depending on the degree of PEGylation. Analysis of cell death showed that complexes of some AgNP variations with siMCL-1 lead to ~70% cell death in the populations that uptake these complexes due to apoptosis.

## 1. Introduction

While researchers are finding more effective ways to treat cardiovascular disease, cancer remains a serious problem for the whole world [1]. One research area is the use of genetic therapy using small non-coding RNAs involved in RNA interference [2]. The best known RNAs from this family are small interfering RNAs (siRNA) and microRNAs (miRNAs) [3,4], both being short-chain nucleotides capable of inhibiting the synthesis of a protein in the cell. These RNAs bind to the corresponding messenger RNA and result in its degradation or irreversible binding. Due to the absence of a direct effect on intranuclear DNA, siRNA exposure does not lead to changes in the host cell genome, leading to a safer approach to genetic therapy when compared with the use of DNA plasmids [5]. siRNA only needs to cross the cell membrane, as RNA interference occurs in the cytosol of the cell. From the point of view of cancer therapy, the suppression of mutant bcl2 genes encoding anti-apoptotic proteins of the same family is an attractive solution [6,7]. Normally, these proteins regulate the processes of apoptosis in the cell; however, in cancer cells, they prevent other cascades from initiating the process of apoptosis associated with the abnormal functioning of the cell.

There are a number of factors that limit the use of siRNAs for clinical purposes. One of the main limitations in the use of siRNAs as therapeutic drugs is their inability to independently penetrate the cell membrane, and unmodified RNAs are also subject to degradation by the action of enzymes when administered intravenously. Therefore, developing an effective method for delivering non-coding RNAs into the cell is an issue. To solve this problem, various types of chemical modifications of RNA are stabilized in the internal environment of the body. However, research so far has primarily focused on the development of biocompatible RNA delivery systems via viral and non-viral carriers. Viral delivery systems are the most effective, but their high carcinogenicity, immunogenicity, and final cost necessitate the development of synthetic vectors [8].

Modified silver nanoparticles (AgNPs) can be used as possible siRNA carriers. These structures have been known for a long time and researchers have managed to develop extremely effective methods of synthesis, control of morphology, and chemical modification. Due to their unique physicochemical properties and biofunctional properties, such as anti-inflammatory, anti-angiogenesis, antiviral, antifungal, and antibacterial activity, AgNPs play an important role in the development and implementation of new biomedical strategies [9]. AgNPs can be successfully used as new nanostructured platforms for the diagnosis and treatment of various types of cancer [10]. AgNPs are promising agents not only for infection control strategies, but also in critical tumor and multidrug resistance approaches due to their broad bioactivity spectrum. There are a number of reports highlighting the identified anti-cancer effects of AgNPs on various human cancer cell lines [11,12]. Currently, a combination of therapy and diagnostics, known as theranostics, is an important, attractive, and challenging approach used for personalized cancer therapy [13]. 

Silver nanoparticles have been less studied than, e.g., gold nanoparticles, but this type of nanostructure has some advantages and, in recent years, more research has been devoted specifically to silver nanoparticles. Modified silver nanoparticles are promising non-viral delivery vehicles, but the issues of overcoming cytotoxic effects by modifying nanoparticles without losing the efficiency of genetic material transfection remain unresolved [14,15,16,17].

Silver nanoparticles have a number of global advantages and disadvantages. Advantages include the ease of synthesis and the comparative cheapness of the volume of nanoparticles. They are also easy to modify, as any markers, linkers, and ligands can be attached to them. The large area of the surface plasmon leads to multitasking of these structures. In the future, modified nanoparticles could be used for tumor imaging and subsequent therapy using photothermal effects. The additional modification of nanoparticles as vectors could be used in a joint therapy involving other drugs. The main disadvantages of silver nanoparticles are their high toxicity and low stability. However, studies show that both toxicity reduction and stability improvement can be achieved with appropriate modifications [18,19]. These include coating nanoparticles with polymers and changing the size and shape of nanoparticles. The search continues for optimal solutions that could not only minimize the cytotoxicity of silver nanoparticles, but also preserve their positive qualities. It can be assumed that a combination of modifications will be more effective, e.g., by combining polymer compounds, fluorophores, and directional ligands (e.g., cell-penetrating peptides) on the surface of nanoparticles, various synergistic effects could be achieved, leading to multitasking [20].

This work concerns the study of the formation of complexes of silver nanoparticles modified with carbosilane dendrons and polyethylene glycol with small interfering RNAs and the influence of these nanoparticles on blood cells. The possibility of delivering siRNAs via modified silver nanoparticles into malignant neoplasm cell lines (two types of leukemia and adenocarcinoma of the cervix) and the targeted effect of siRNAs of the group aimed at silencing the bcl2 family were studied. The effect of polyethylene glycol modification (PEGylation) on the ability of nanoparticles to bind miRNAs into complexes and deliver them to cells in vitro was analyzed.

## 2. Results

### 2.1. AgNP-siRNA Complexation

#### 2.1.1. Changes in the Secondary Structure of RNA in the Presence of AgNP

CD spectroscopy was used to study the effect of nanoparticles on the secondary structure of siRNA. The CD spectra of siRNAs (Figures S1–S9) are typical of the A-form of the RNA secondary structure, with characteristic peaks at ~210 nm and ~260 nm. The molar ellipticity of siRNAs decreased with increasing AgNP concentration in almost all cases (except for 1bAg and 1cAg). In most cases, the negative peak of the CD spectra disappeared completely or almost completely, indicating significant changes in the secondary structure of RNA with high doses of AgNP. 

**Figure 1:**
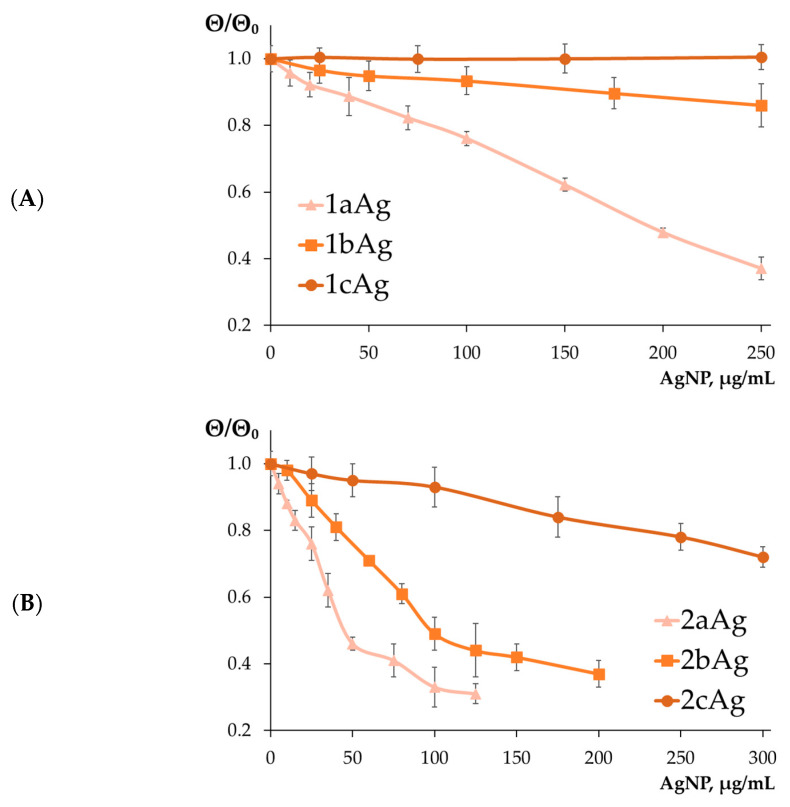

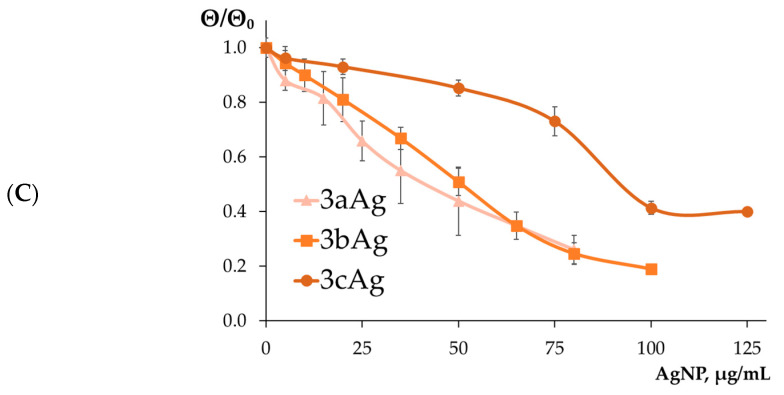
Change in relative molar ellipticity of siMCL-1 at λ = 260 nm in the presence of AgNP with (**A**) 1st-generation, (**B**) 2nd-generation, and (**C**) 3rd-generation surface dendrons. Concentration of siRNA = 1 μM; T = 25 °C. Data are presented as relative molar ellipticity (**Θ/Θ_0_**), mean ± SD, *n* = 4.

The positive peak of the CD spectra is significant for assessing changes in the secondary structure. Figure 1 shows changes in the relative molar ellipticity of siRNAs treated with AgNPs. Changes in the secondary structure increased for higher generations, but regardless of the generation of surface dendrons, changes in the secondary structure significantly leveled off at a high degree of PEGylation. However, a limiting change in the intensity of the molar ellipticity peak could be observed, associated with nanoparticle absorption rates in the ultraviolet region with increasing concentrations. In many cases, this interfered with data collection at concentrations above 200 µg/mL.

#### 2.1.2. Determination of the Formation of AgNP-siRNA Complexes

**Figure 2:**
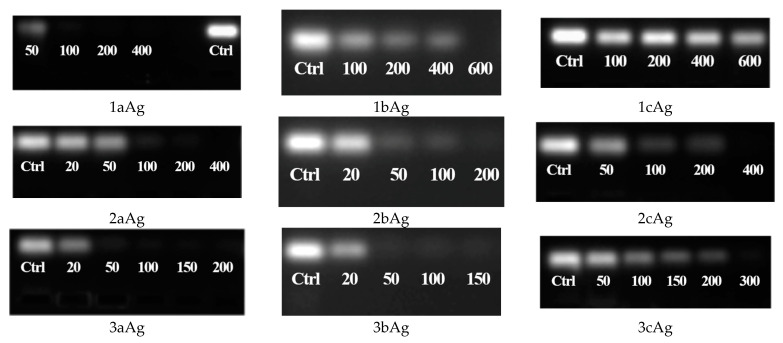
Gel electrophoresis of AgNP-siRNA complexes at varying AgNP/siRNA mass-molar ratios. Numerical values on the images are given in µg/mL of corresponding AgNP. The caption under the image means the corresponding nanoparticle. Concentration of siRNA (siMCL-1) = 1.5 μM; incubation time, 20 min; T = 25 °C. “Ctrl” is non-treated siRNA control well for signal level comparison.

In order to establish whether AgNPs form stable complexes with siRNAs, a series of experiments used the method of one-dimensional agarose gel electrophoresis. Figure 2 presents gel electrophoresis images with AgNP-siRNA complexes. The images show that a high degree of PEGylation of nanoparticles sharply reduced the binding ability, especially in the case of AgNP-G1. 

It was also shown (Figure S10 in the Supplementary Materials) that the resulting complexes were able to remain stable in PBS for at least 5 days.

#### 2.1.3. Estimation of the Surface Charge of AgNP-siRNA Complexes

**Figure 3:**
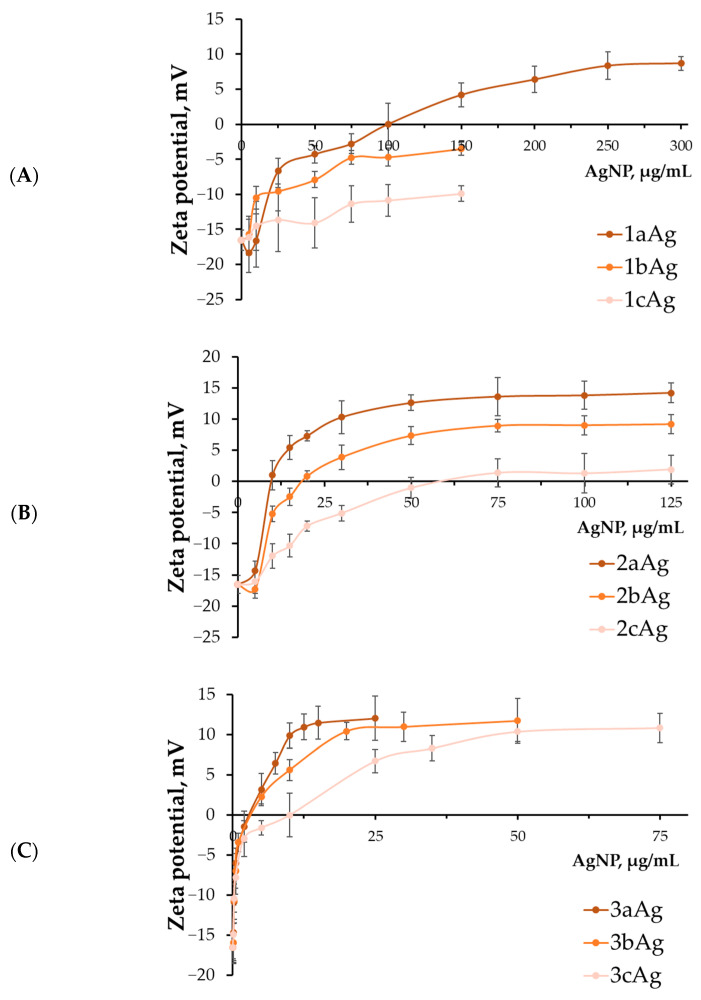
Change in surface charge of complexes AgNP-siMCL-1 with (**A**) 1st-generation, (**B**) 2nd-generation, and (**C**) 3rd-generation surface dendrons. Concentration of siRNA = 0.5 μM; T = 25 °C. Data were obtained based on mobility of particles in an electric field by dynamic light scattering, mean ± SD, *n* = 3.

Zeta potential data are consistent with previous findings (Figure 3). AgNP-G1 weakly bound siRNAs into complexes, and the resulting 1bAgNP and 1cAgNP complexes had a negative charge, which can subsequently become an obstacle to cell penetration. 1aAg still formed positive complexes with a saturation plateau at 8.7 ± 1.0 mV. Complexes based on AgNP-G2 were also formed depending on the degree of PEGylation. In this case, a plateau was observed at approximately equal concentrations; however, the plateau values increased with a decrease in the PEG ratio. A different picture was observed with AgNP-G3. In all cases, the plateau reached ~11.5 mV; however, higher concentrations were required to reach the plateau values as the PEG ratio increased.

#### 2.1.4. Hydrodynamic Size of AgNP-siRNA Complexes

**Figure 4:**
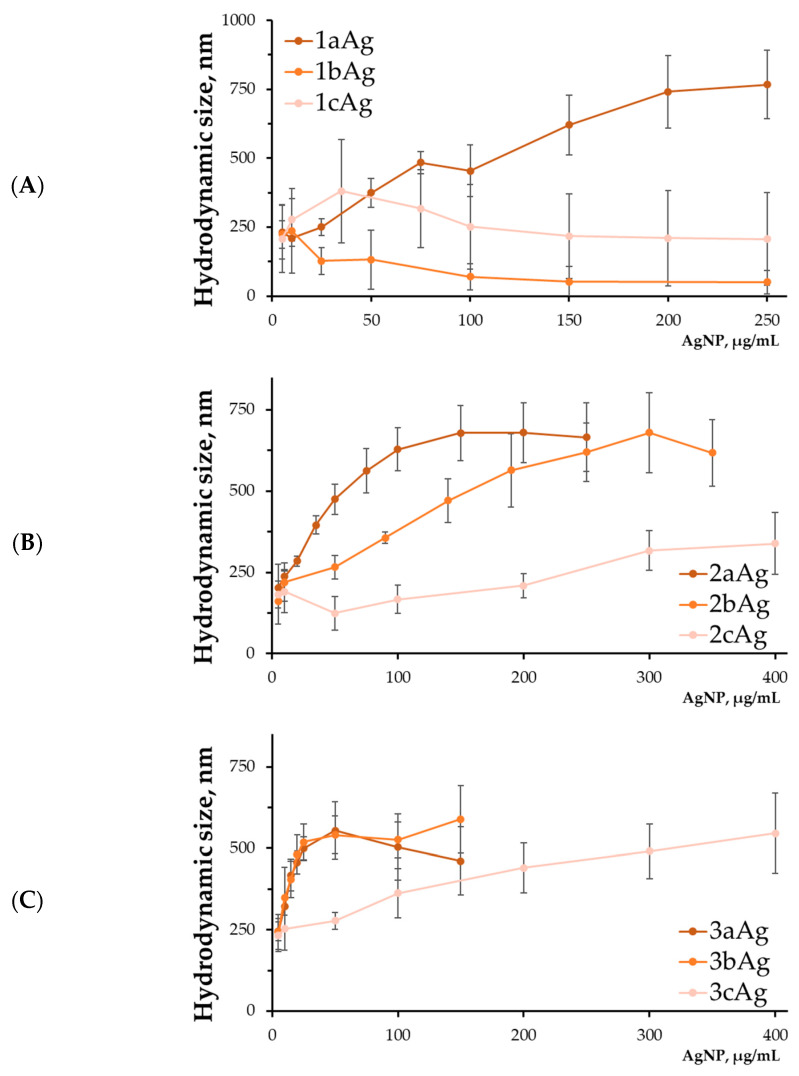
Hydrodynamic size of complexes AgNP-siMCL-1 with (**A**) 1st-generation, (**B**) 2nd-generation, and (**C**) 3rd-generation surface dendrons. Concentration of siRNA = 0.5 μM; T = 25 °C. Data were obtained by dynamic light scattering, mean ± SD, *n* = 3.

Determination of the hydrodynamic size of the complexes demonstrated the significant size of the resulting structures (Figure 4). Relatively small sizes of the complexes were obtained in the case of 1bAg and 1cAg and amounted to ~50 and ~200 nm, respectively, in the plateau region. However, these complexes had a negative surface charge and did not form stable complexes. In other cases, the sizes of the complexes reached ~500–750 nm. Large scatter in the observed data and a high level of polydispersity indicated that the complexes were highly heterogeneous in size and probably had a loose structure. The previously discovered nature of the dependence on the degree of PEGylation was observed, namely, the higher it was, the weaker the degree of binding and the higher the required concentrations of nanoparticles.

### 2.2. Toxicity of Silver Nanoparticles for Blood Cells

#### 2.2.1. Hemotoxicity

**Figure 5:**
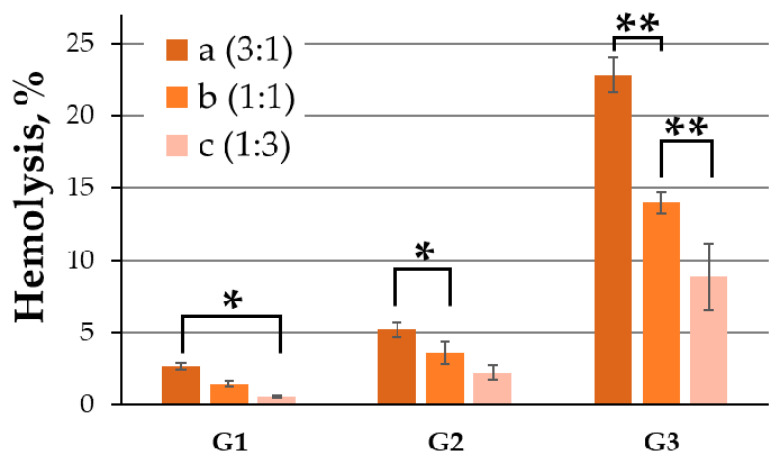
Hemolysis induced by AgNPs after 24 h of incubation. Concentration of all AgNPs equal to 50 µg/mL. Data are presented as percentage of hemolysis, mean ± SD, *n* = 4. Statistical significance between AgNP within a group (at * *p* < 0.01, at ** *p* < 0.0002) was estimated by the post hoc Newman–Keuls test.

The concentration of free hemoglobin was used to determine the percentage of hemolysis in the RBC fraction. The data indicated the rise in toxicity with an increase in the generation of surface dendrons and a decrease in the degree of PEGylation. Figure 5 shows hemolysis data after 24 h under the action of silver nanoparticles at a concentration of 50 μg/mL. Data for 2 and 24 h and 10 and 25 µg/mL are shown in Figure S34 in the Supplementary Information. Considering that much lower concentrations will be used in future delivery, such toxicity values should be considered acceptable, along with other factors that are discussed below.

#### 2.2.2. PBMC Inhibition

**Figure 6:**
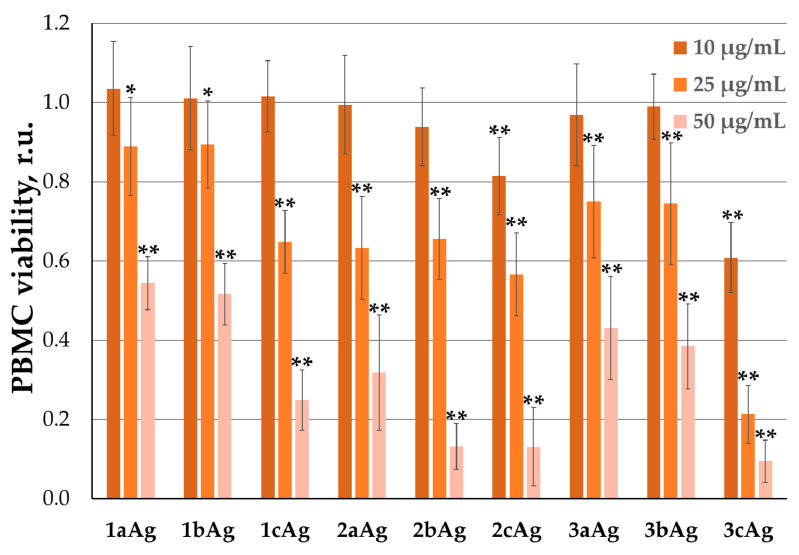
Inhibition of lymphocyte viability by AgNPs after 72 h of treatment. Data are presented as percentage of viability of control cells, mean ± SD, *n* = 6. Statistical significance between treated sample and control (at * *p* < 0.05, at ** *p* < 0.0001) was estimated by the post hoc Newman–Keuls test.

The study of PBMC inhibition showed a different situation (Figure 6). Here, viability was influenced not by the generation of surface dendrons, but by the degree of PEGylation. While at low concentrations (10 µg/mL), most nanoparticles did not have a significant toxic effect, 2cAg and 3cAg (dendron/PEG ratio 3:1) resulted in a reduction in viability of up to 80% and 60%, respectively. This may be evidence of some form of immune response to short PEG chains.

### 2.3. Cellular Uptake of Nanoparticle Complex

In order to establish the optimal concentrations of AgNPs for further use in siRNA delivery, four concentrations of AgNPs of all types and one concentration of siRNAs (100 nM) were tested. (Full curves for the three cell lines studied are presented in the supplementary information in Figures S11–S28). Measurements were also carried out for two incubation times: 3 and 24 h, to observe the dynamics. The data indicated that AgNP-G1 did not deliver siRNA into the cell. The maximum observed cellular uptake was 4% for the HL60 cell line after 24 h of incubation (Figure S18). Therefore, this group of nanoparticles was excluded from consideration in further experiments related to the delivery of targeted siRNA.

**Figure 7:**
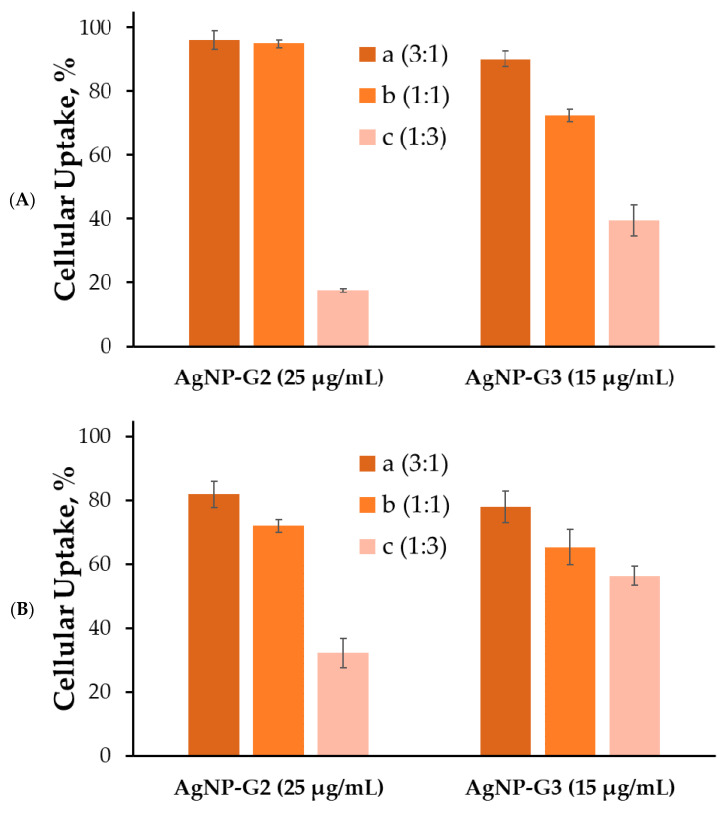
Cellular uptake of complexes with AgNP and siRNA (ntRNA-FAM, 100 nM) complexes with optimal concentrations of AgNP in HL60 cells after (**A**) 3 h and (**B**) 24 h of incubation. Optimal concentrations of AgNP were 40 µg/mL for AgNP-G1; 25 µg/mL for AgNP-G2; 15 µg/mL for AgNP-G3. Data were obtained based on fluorescence intensity from FAM-labeled RNA by flow cytometry. Data are presented as percentage of cellular uptake, mean ± SD, *n* = 4.

**Figure 8:**
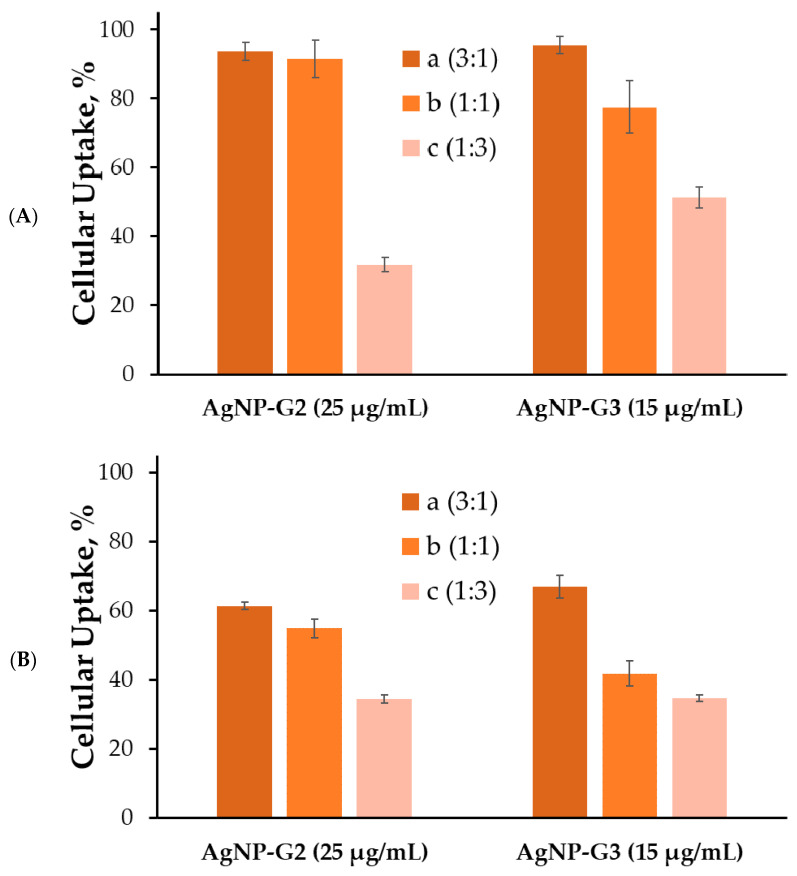
Cellular uptake of complexes with AgNP and siRNA (ntRNA-FAM, 100 nM) in CEM-SS cells after (**A**) 3 h and (**B**) 24 h of incubation. Data were obtained based on fluorescence intensity from FAM-labeled RNA by flow cytometry. Data are presented as percentage of cellular uptake, mean ± SD, *n* = 4.

For some reason, HeLa also turned out to be weakly susceptible to the uptake of complexes based on modified AgNPs; the level of internalization did not exceed 5–10% (Figures S12–S16). However, in the two leukemia-associated cell lines studied, AgNP delivered FAM-labeled siRNA surprisingly well. In both HL60 (Figure 7) and CEM-SS (Figure 8), 2aAg and 2bAg achieved internalization in almost 100% of the cell population after 2 h. 2cAg delivered genetic material noticeably weaker after 2 h. If the previously mentioned nanoparticles reduced the level in the population, then the content of 2cAg gradually increased. A similar situation was observed in the AgNP-G3 group and the effect of PEGylation was more pronounced. The maximum internalization in HL60 cells after 2 h was 90–95% for 3aAg and did not decrease after 24 h of incubation. Apparently, CEM-SS cells were more efficient in removing or degrading foreign complexes, as after 3 h, levels of internalization were quite similar to those of HL60 cells; however, the proportion of fluorescent cells decreased markedly in these cells after 24 h of incubation.

### 2.4. Cytotoxicity Effects of AgNPs and Their Complexes with siMCL-1

Using the MTT test, we described the cytotoxicity curves for three cell lines (Figures S29–S31). A characteristic and important effect was the detection of significant HeLa proliferation in the presence of AgNP (Figure S31). Cellular activity increased up to 4.5 times compared to the control for AgNP-G2 and G3. For AgNP-G1, the dose–response curve retained its usual form. The dependence on the degree of PEGylation indicated that surface dendrons were primarily involved in the process of increasing proliferative activity.

For the other two cell lines, no increase in viability was observed, though, in some cases, statistically significant undulating behavior of the curve can be observed, which may indicate much more subtle, but similar to HeLa, mechanisms. It is important to note that PEGylation played little to no role for AgNP-G3, as the dose–response curves in some cases coincided within the margin of error.

**Figure 9:**
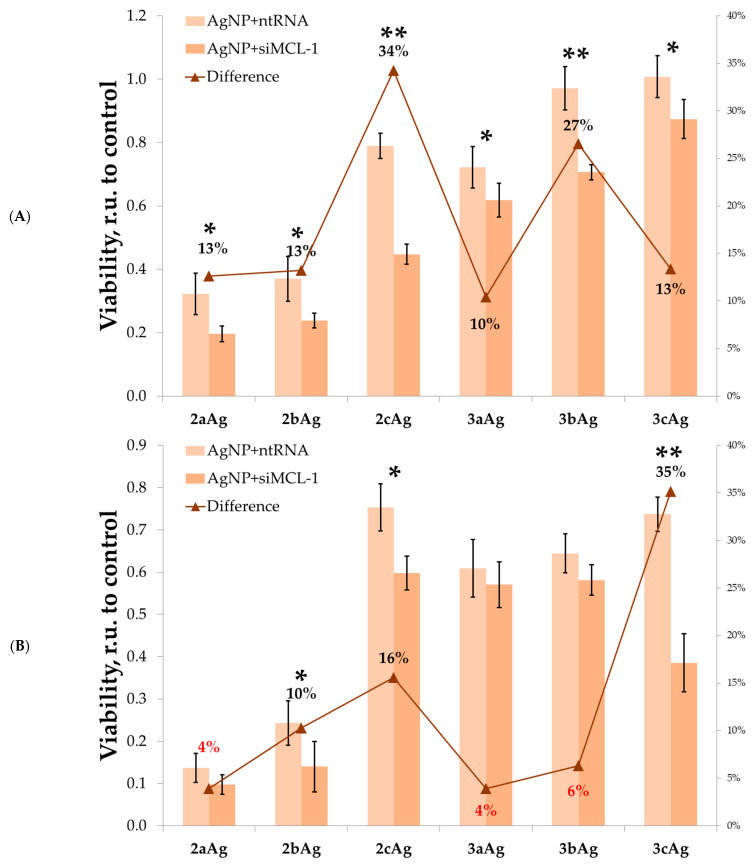
Viability of (**A**) HL60 and (**B**) CEM-SS treated with complexes of AgNP with target siRNA (siMCL-1) and control sequence (ntRNA) after 72 h of incubation. AgNP concentrations were equal to 25 µg/mL for AgNP-G2; 15 µg/mL for AgNP-G3; siRNA concentration was equal to 100 nM. The broken line (-▲-) represents the difference between AgNP complexes with control and with target siRNAs. Data were obtained by Alamar Blue test. Data are presented as relative units to control of untreated cells, mean ± SD, *n* = 4. Statistical significance between treated sample and control sample (at * *p* < 0.05, at ** *p* < 0.001) was estimated by the post hoc Newman–Keuls test. Values with an unreliable difference are marked in red.

Next, we tested the difference in effects from complexes with target (siMCL-1) and control (ntRNA) siRNA (Figure 9). Complexes with non-targeted RNA were slightly less toxic compared to AgNP per se. Complexes with targeted siRNA in all cases showed significantly greater toxicity. 2cAg for HL60 cells and 3cAg for CEM-SS cells had the greatest effect. In these cases, the survival difference between complexes based on targeted and non-targeted siRNAs was 34% and 35%, respectively. In addition, a significant effect, amounting to 27% of the difference, was observed when using 3bAg against HL60 cells.

### 2.5. Assessment of Cell Death Due to Exposure to AgNP-siRNA Complexes

**Figure 10:**
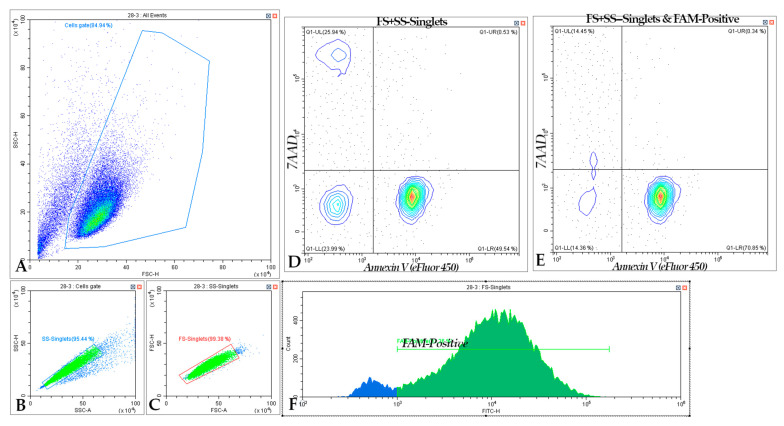
An example of cell gating to determine populations by cell death by flow cytometry. The process of building gates contained the following stages: (**A**) choosing a population with cutting out trash events; choosing of singlet events in (**B**) side and (**C**) forward scattering channels. Analysis of the distribution of events along the 7AAD (PerCP) and Annexin V (PB) channels in single cells of (**D**) the entire population and (**E**) FAM-positive cells, obtained based on the combined population of singlet and FAM-positive cells (**B**,**C**,**F**). FAM-positive cells were determined by (**F**) fluorescence intensity relative to the autofluorescence control of unstained cells.

The levels of cell death under the action of the studied silver nanoparticles and their complexes with targeted and non-targeted siRNAs were determined. An example of flow cytometry gating is shown in Figure 10. Apoptosis was measured using labeled protein Annexin V; this protein is able to bind to phosphatidylserine on the outer side of the cell membrane. This marker is often used as evidence of the initiation of apoptosis in the cell, as the transfer of phosphatidylserine to the inner side of the membrane is an ATP-dependent process. Necrosis was determined by the dye 7AAD, which cannot voluntarily pass into the cell through an intact membrane. In the case of damage and pores on the cell, a sign of necrotic processes, 7AAD enters the cell and intercalates double-stranded DNA in cytosine-guanine-rich regions. Thus, single positive events for Annexin V are defined as early apoptosis; single positive events on 7AAD are identified as early necrosis. Double-positive events, where cells fluoresce in two channels, are defined as “later-stage apoptosis”. It should be noted that the nature of doubly positive events cannot be reliably determined. Both necrotic cells with pores sufficient for the penetration of Annexin V and later apoptotic cells, the membrane of which is damaged as a result of vesiculation, are present. During sample preparation, a part of apoptotic vesicles and necrotic cells may be lost, so the population, especially of necrotic cells, will be reduced compared to the real situation [21,22].

A number of experiments (not presented in the article) were performed, resulting in the findings that the most optimal time for measurement was the period after 48 h of incubation with nanoparticles. During this period of time, the antisense strand of the target siRNA has time to leave the complexes and trigger RNAi mechanisms in the cell and the FAM label attached to the sense strand has not yet undergone intracellular degradation, so it possible to analyze the population with siRNA uptake.

**Figure 11:**
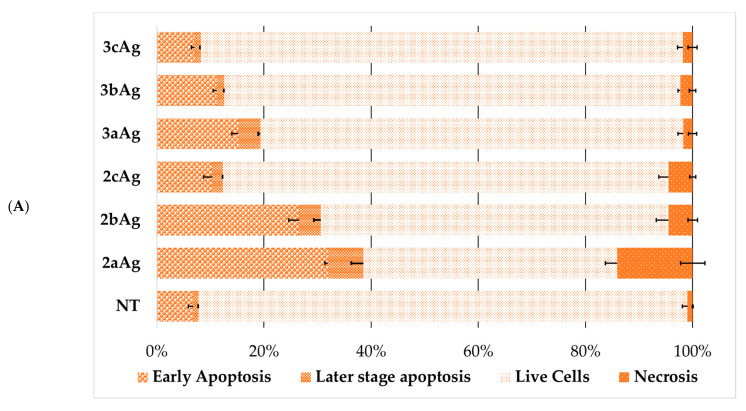

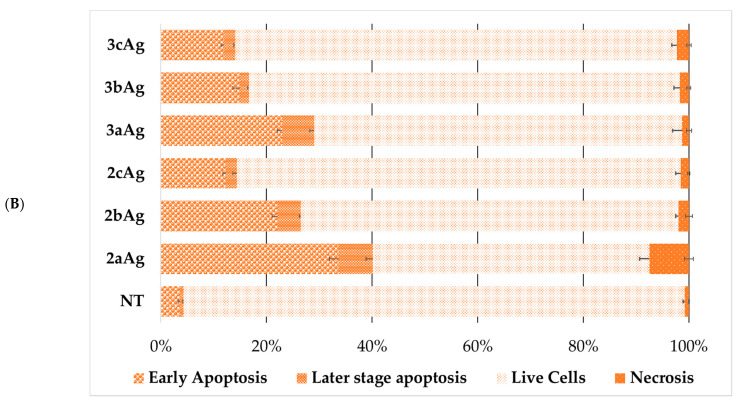
Distribution of (**A**) HL60 and (**B**) CEM-SS cells by types of cell death among the entire population of cells after 48 h of incubation treated with AgNP. AgNP concentrations were equal to 25 µg/mL for AgNP-G2; 15 µg/mL for AgNP-G3; siRNA concentration was equal to 100 nM. Data were obtained by flow cytometry. In each repeat, 100,000 events were collected and analyzed.

Initially, we tested the effect of silver nanoparticles on the mechanisms of cell death. Figure 11 shows the distribution of HL60 and CEM-SS cell populations. There was a regular decrease in the apoptosis fraction with an increase in the PEG ratio. An important feature is the fact that nanoparticles trigger cell death primarily by the mechanism of apoptosis. This type of cell death is the safest for organisms, as cellular material is packed into vesicles and subsequently removed by the immune system. A sufficient degree of necrosis was observed only when HL60 cells were treated with 2aAg silver nanoparticles. Here, the contribution of necrosis was 14.1 ± 2.3%.

**Figure 12:**
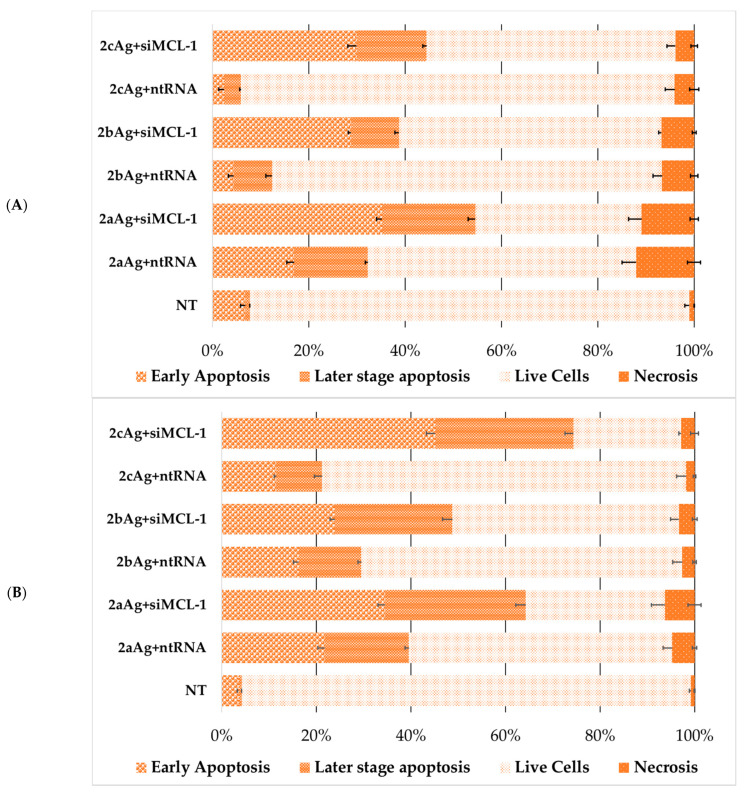
Distribution of (**A**) HL60 and (**B**) CEM-SS cells by types of cell death among the entire population of cells after 48 h of incubation treated with complexes AgNP-G2 with target (siMCL-1) and control (ntRNA) RNA. AgNP-G2 concentrations were equal to 25 µg/mL; siRNA concentration was equal to 100 nM. Data were obtained by flow cytometry. In each repeat, 100,000 events were collected and analyzed.

**Figure 13:**
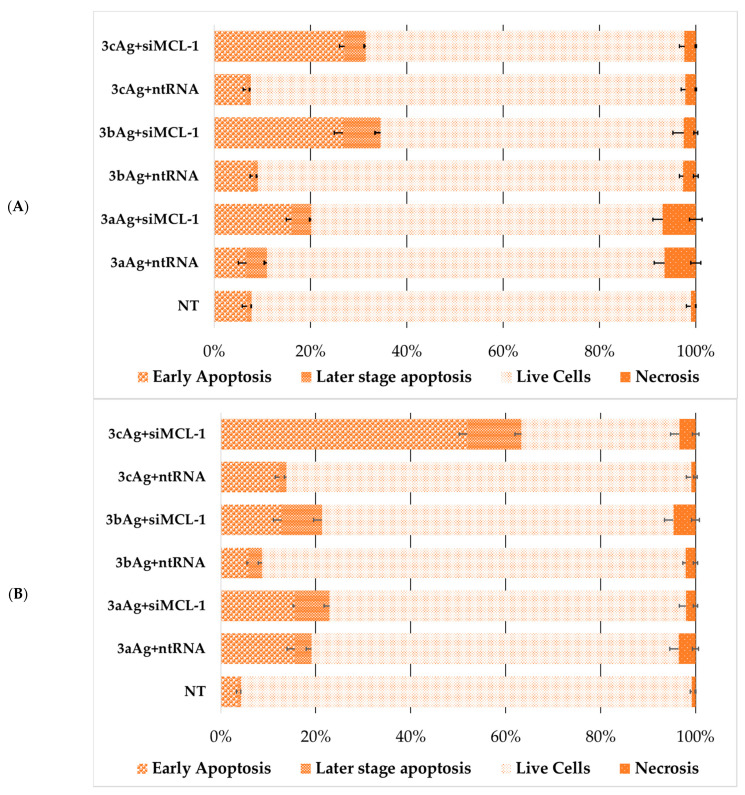
Distribution of (**A**) HL60 and (**B**) CEM-SS cells by types of cell death among the entire population of cells after 48 h of incubation treated with complexes AgNP-G3 with siMCL-1. AgNP-G3 concentrations were equal to 15 µg/mL; siRNA concentration was equal to 100 nM. Data were obtained by flow cytometry. In each repeat, 100,000 events were collected and analyzed.

At the next stage, the death of cells treated with complexes with target and control siRNA was analyzed (Figure 12 and Figure 13). The change in the characteristics of the distribution of cell death for AgNP-ntRNA complexes was immediately noticeable and the number of cells that were in the stage designated as “later stage apoptosis” increased. This may mean membrane damage by the action of complexes, incorporation of complexes into membranes, and other damage to the lipid layer. Complexes based on target siRNA significantly led to an increase in cell death in all cases by the mechanism of early apoptosis and the later stage of apoptosis. Moreover, the necrotic fraction in the population did not change between the target and control siRNA.

The distribution of populations by cell death in the population of FAM-positive cells, i.e., cells where complexes with siRNAs significantly internalized, was interesting. Previously, we found that FAM on the sense strand of siRNA does not affect the distribution of the cell death population, so this analysis is acceptable. This primarily explains the observed difference between the level of cellular uptake of the complexes and the difference in cytotoxic effect between targeted and non-targeted siRNAs.

**Figure 14:**
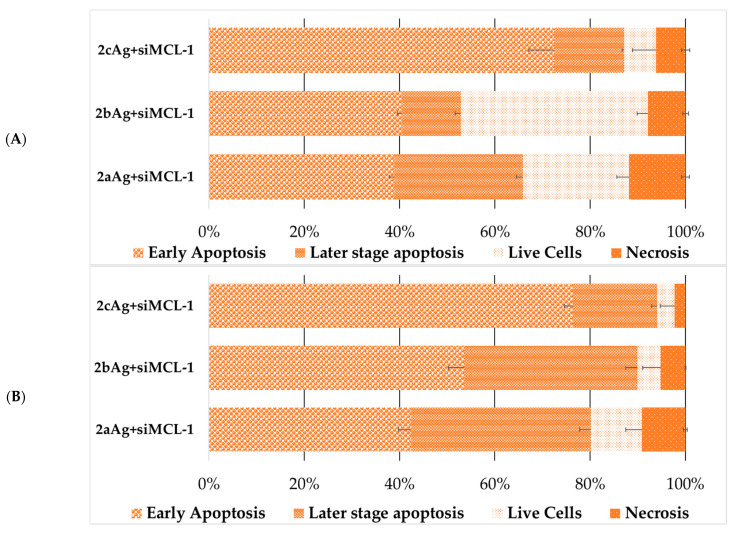
Distribution of (**A**) HL60 and (**B**) CEM-SS cells by types of cell death among the FAM-positive cells after 48 h of incubation treated with complexes AgNP-G2 with target (siMCL-1) and control (ntRNA) RNA. AgNP-G2 concentrations were equal to 25 µg/mL; siRNA concentration was equal to 100 nM. Data were obtained by flow cytometry. In each repeat, 100,000 events were collected and analyzed.

**Figure 15:**
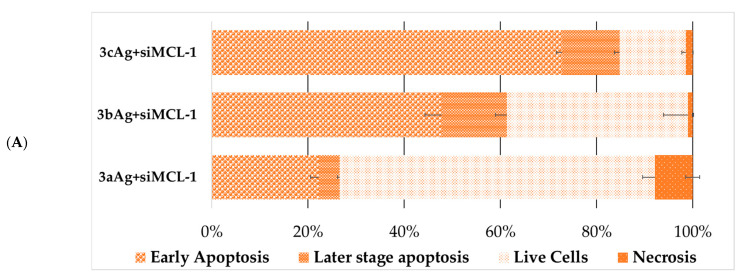

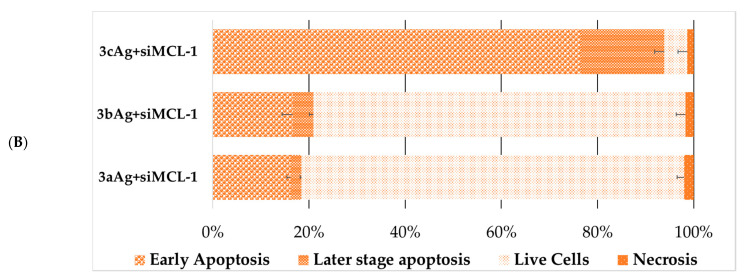
Distribution of (**A**) HL60 and (**B**) CEM-SS cells by types of cell death among the FAM-positive cells after 48 h of incubation treated with complexes AgNP-G3 with siMCL-1. AgNP-G3 concentrations were equal to 15 µg/mL; siRNA concentration was equal to 100 nM. Data were obtained by flow cytometry. In each repeat, 100,000 events were collected and analyzed.

As expected, the number of living cells was sharply lower within the population of FAM-positive cells after treatment with complexes with targeted siRNA (Figure 14 and Figure 15), when compared to the general population. Complexes with control siRNA did not significantly differ from the general population (Figures S32 and S33). The nature of the effect was very different both between cell lines and between the generation of surface dendrons and the degree of PEGylation. Therefore, the contribution of apoptosis of the HL60 cells treated with 2aAg and 2bAg was approximately equal, while the later-stage apoptosis relative to 2aAg was almost twice as high than that of 2bAg. The effect of AgNP-G2 on CEM-SS cells appeared to be less dependent on PEGylation. Here, the population of living cells overall was quite small; however, there was a smaller ratio of apoptosis to later-stage apoptosis for 1cAg. AgNP-G3 on the HL60 line behaved predictably, as, with increases in the PEG ratio, the number of apoptotic cells in the population increased. At the same time, CEM-SS cells were not susceptible to complexes based on 3aAg and 3bAg, and 3cAg-siMCL-1 complexes led to almost complete cell death by apoptosis within the population.

## 3. Discussion

Currently, the prospect of using silver nanoparticles as multifunctional agents in cancer therapy is being researched [23,24]. In this work, AgNPs were chosen as an object of study as a potential non-viral vector, due to unique optical characteristics and the larger area of the plasmon field when compared to gold nanoparticles. These properties distinguish them as candidates for creating biosensors, substrates for fluorescent absorption, and photo-controlled delivery systems [25,26,27]. Plasmon resonance of silver nanoparticles can potentially be used in plasmonic photothermal therapy (PPTT) [28,29]. In the future, the combined use of proapoptotic siRNAs and PPTT against tumors may contribute to more effective therapy, while proapoptotic siRNAs turn off antiapoptotic proteins, raising the temperature by PPTT, which may contribute to more efficient initiation of apoptosis in tumor cells. However, the possibility of using PPTT for modified nanoparticles requires additional research [29,30,31].

In the context of this work, we evaluated the fundamental possibility of using AgNPs modified with carbosilane dendrons and PEG. Previously, we studied dendronized AgNPs; however, their cytotoxic effects were quite high, despite the high efficiency of internalization [32]. It was assumed that PEG modification would reduce toxicity with some loss of internalization efficiency. This assumption is based on the fact that dendronized and simultaneously PEGylated silver nanoparticles have a lower total surface charge compared to dendronized non-PEGylated AgNPs due to their physically limited number of binding sites on the surface. In turn, the electric charge density also decreases. The second assumption follows that nanoparticles with a high degree of PEGylation will retain genetic material less than nanoparticles with a high degree of dendronization, meaning that they will release it more efficiently in the cytoplasm, in cases of release by the “proton sponge” effect [33].

The work was divided into three stages: (i) determination of the binding parameters of nanoparticles and siRNAs using molecular biophysics methods; (ii) study of the cytotoxic effects of AgNPs on blood cells; (iii) evaluation of the effectiveness of AgNP-siRNA complexes against tumor cell lines in vitro.

From molecular studies, it follows that dendronized and PEGylated silver nanoparticles are able to bind siRNA according to the following regularities: stronger binding was observed with (i) increased generation of surface dendrons and (ii) high ratio of dendrons to PEG. An increase in the degree of PEGylation leads to a deterioration in the binding of nanoparticles to the genetic material, as a result of which the concentrations required for complete binding of the material become noticeably higher [34]. Possible reasons for this effect have been suggested above.

It is indicative that 1bAg and 1cAg nanoparticles with an equal and high ratio of PEG to dendrons, respectively, demonstrated a minor level of interaction with genetic material. The resulting complexes had a small size but a negative surface charge, which became an obstacle to their uptake in all studied cell-lines. As a result, AgNP-G1 was no longer used or only screened for studies of complex cytotoxicity and cell death assays. 

AgNP-G2 and AgNP-G3 group results were more optimistic. According to data obtained by biophysical methods, these nanoparticles bind genetic material weakly at high PEG ratios but do not lose this ability to the same extent as AgNP-G1. At the same time, this behavior differs significantly between the AgNP-G2 and AgNP-G3 groups. Therefore, according to the zeta potential method, AgNP-G3 in all variants reached the same value of complex plateau saturation at significantly different concentrations. The plateau concentrations for AgNP-G2 were close but the absolute values of the plateau in mV decreased with an increase in the ratio of PEG/dendron. For 2cAg, the zeta potential values turn out to be close to zero, which, as discussed below, significantly reduces its effectiveness during in vitro internalization.

The maximum hydrodynamic size of the complexes turned out to be quite large. For AgNP-G2, with the exception of 2cAg, the size of the complexes was ~650 nm, while for the AgNP-G3 group, sizes varied within 500 nm. Even though the hydrodynamic size, as a rule, is 1.5–2.0 times the actual size of the complexes themselves without a hydration shell [35], the obtained values are still quite large when compared to the sizes of the complexes that are optimal for delivery. It is known that particles up to 100 nm are preferred for therapeutic purposes because they enter cells via clathrin-mediated endocytosis. For larger particles, caveola-mediated internalization occurs with this mode being predominant for particles up to 500 nm [36,37,38,39]. 

It was found that optimal delivery concentrations of AgNPs fall on areas close to the minimum concentrations of genetic material binding. The optimum does not depend on the degree of PEGylation of silver nanoparticles. This is primarily due to the size of the resulting complexes: the lower the concentration of silver nanoparticles relative to siRNA, the lower the complexes formed by these nanoparticles, according to the data obtained by dynamic light scattering. Accordingly, with the rise in concentration, the larger the complexes will be and more difficult it will be to internalize into the cell. At lower concentrations, stable complexes are not yet formed. However, there are reports that sufficiently large complexes can be efficiently taken up by cells. In addition, it seems that the order of magnitude of the optimal delivery concentration is close for similar compounds with a rigid metal core [40,41].

An analysis of the effect of silver nanoparticles on blood cells showed that PEGylation significantly reduces the hemolytic effect. In earlier work, we used similar non-PEGylated silver nanoparticles [32] and showed that, after treatment with 20 μM (~50 μg/mL) 3Ag, hemolysis exceeded 80% after 24 h of incubation. At the same time, similar structures, even with a minimal (3:1) degree of PEGylation, reduced this indicator to 22.8 ± 1.2%. For PEGylated AgNPs, there is a tendency to increase the level of hemolysis with an increase in the relative number of surface dendrons. However, there are a number of observations regarding the low toxicity of the proposed nanoparticles. First, the maximum concentrations used were at least twice the optimal delivery concentrations. For example, for AgNP-G2, the optimal concentration was taken to be 25 µg/mL. The value of hemolysis for the most toxic 2aAg in this case was only 3.4 ± 1.1%. Secondly, the model experiment assumed the absence of albumin in the solution, while nanostructures with a positively charged surface form the so-called “protein corona” in the bloodstream, sharply reducing the toxicity of similar nanostructures [42,43,44]. Finally, the complexes themselves normally exhibit significantly less toxicity to blood cells and the selected model experiment assumed limiting blood saturation with silver nanoparticles, toward complexes being completely destroyed in the blood and all nanoparticles being in a free state. Additionally, PBMCs were strongly affected by modified silver nanoparticles; there was a sharp drop in the viability of PBMCs with an increase in AgNP concentration. Graphs showed that PEG is the main contributor, as, with an increase in the degree of PEGylation, toxic effects become more and more noticeable. However, in this case, the optimal delivery concentrations did not have such a detrimental effect. Thus, for AgNP-G2, viability remains at the level of 60%. In the future, for immunophenotyping methods, it would be necessary to investigate the mechanisms of toxicity and the immune response of PBMC more thoroughly to determine which fractions of nanoparticles are affected acutely. As the previously studied PEGylated and dendronized gold nanoparticles and dendronized silver nanoparticles [32,34] did not show such acute effects on PBMCs, it can be assumed that both PEG and the silver core are involved in the toxicity mechanism. PEG enhances the cumulative effect of silver nanoparticles, as described in the literature [29,45,46]. Finally, experiments were carried out on platelet aggregation in the presence of the investigated silver nanoparticles (data not shown). In a series of experiments, no effects were found promoting or preventing aggregation, with the conclusion that the studied nanoparticles are inert with respect to platelets.

An analysis of the toxic effect of nanoparticles per se and their complexes leads initially to somewhat contradictory results. On the one hand, AgNP with a high degree of PEGylation (i) weakly binds genetic material, according to data obtained by molecular biophysics, and (ii) is weakly internalized into cells. On the other hand, the decrease in viability is most pronounced in cells treated with complexes of targeted siRNA and AgNP with a dendron/PEG ratio of 1:3. This contradiction is resolved by the fact that high-PEGylated nanoparticles probably have a prolonged effect, while AgNP with dendron/PEG ratios of 1:1 and 3:1 rapidly accumulate in cells after 3 h and after 24 h, and their presence in the cells decreases. At this time, the level of internalization of highly PEGylated AgNPs either increased or decreased slightly over time. In addition, cells continued to proliferate during the experiment, partly explaining the decrease in the level of internalization of the remaining nanoparticles. AgNPs with a high content of PEG are accumulated by cells gradually and are possibly less prone to cellular mechanisms of excretion and utilization. An important confirmation was the observation of the population of FAM-positive cells. It was shown that within this population, the percentage of cells in the state of apoptosis is noticeably higher than in the general population, indicating that these cells are the main source of the Annexin V signal in the general population of analyzed cells; this is confirmed by obtained data. Highly PEGylated nanoparticles have a lower ability to internalize; nevertheless, when they enter the cell, they significantly reduce cell viability due to apoptosis. They have the same efficiency as equally dendronized and PEGylated nanoparticles, but are generally less toxic. Apparently, the silver core itself also plays a key role as modified silver nanoparticles induce cell death predominantly through the mechanism of apoptosis. Previous studies have shown that silver nanoparticles can induce apoptosis and cytotoxic effects by different mechanisms in various cells, including human cervical cancer and acute monocytic leukemia [47,48,49]. The cytotoxic effect of AgNP appears to be primarily a result of the formation of reactive oxygen species (ROS) [50,51]. Simultaneously, complexes based on the same nanoparticles lead to cell death by other mechanisms as the percentage of doubly positive events (the later stage of apoptosis) is higher than in the case of AgNP per se. It is possible that there is damage or thinning of the membrane, so Annexin V and 7AAD are able to enter the cell at the same time, while the cell does not yet lose its shape and is of sufficient size not to register as debris particles. Unfortunately, it is not possible to determine whether there is any synergistic effect from the use of silver nanoparticles in combination with the proapoptotic siRNAs used in this work. However, taking into account the generalized data, it can be assumed that at least part of the AgNPs is involved in the cellular mechanism after the release of siRNAs from the complexes.

To confirm gene silencing, an attempt was made to perform PCR-RT (data not shown); however, despite differing mean values compared to the control, the data obtained had too high standard deviation values when cells were treated with AgNP complexes, making it impossible to form a reliable conclusion about the action of the complexes. As AgNPs can initiate DNA damage [52], and samples after the washing protocol may retain some amounts of nanoparticles that were previously internalized into cells, it is likely that cDNA damage occurs in samples with a disruption of the further amplification process.

It should be noted that all of the above applies to a greater extent in leukemic cell lines. There are a number of significant problems with regard to HeLa epithelial cells. Excessive proliferation in a certain range of AgNP concentrations is an alarming signal. A number of screening experiments on other cell lines were carried out (HepG2, HT29, and MCF-7), with findings that 2 out of 4 cancer cell lines of epithelial origin (MCF-7 and HeLa) tend to increase proliferation in the presence of a low concentration of investigated nanoparticles. It can be assumed that some cells of the tumor epithelium have protective mechanisms leading to pronounced proliferation, but what exactly causes such a reaction is unknown. We see that proliferative activity decreases with increasing levels of PEGylation, but in our previous studies, where nanoparticles were modified only with dendrons, no such regularity was observed. It is possible that a joint action also takes place here, as the toxic effects that showed previously and depended on the object under consideration could be initiated by both dendrons and PEG. It is difficult to find data in the literature that could clarify the situation, but it is important to report this. The second problem associated with a low level of internalization seems to be specific only for HeLa cells, as, in other cases, AgNPs were taken up by the above epithelial tumor lines at an acceptable level. 

Therefore, modified AgNPs are promising non-viral vectors for the delivery of small RNAs to leukemic cell lines in vitro. The toxicity and possible immunogenicity of the studied nanoparticles remain an unsolved problem. Despite the significantly lower erythrocyte toxicity values, the problem of PBMC toxicity persists, but here, the toxicity mechanisms appear to be related to PEG rather than cationic dendrons. It is assumed that similar use of other polymers can give comparable results and completely avoid toxic effects on blood cells. The problem of the size of the complexes could be solved by varying the size of the silver core, using other combinations of dendrons and polymers and reducing the total number of binding sites on the nanoparticle surface, etc. Nevertheless, it is difficult to predict how any proposed nanosystem will behave, especially as the core size changes. Our further research goal is to consider the possibility of using modified AgNPs for plasmonic photothermal therapy in combination with genetic therapy based on RNA interference.

## 4. Materials and Methods

### 4.1. Small Interfering RNA

Non-fluorescent and FAM-labeled siMCL-1 (sense 5′-GGA CUU UUA UAC CUG UUA UdTdT-3′; antisense 5′-AUA ACA GGU AUA AAA GUC CdTdT-3′) and ntRNA (1st chain 5′-AAT TCT CCG AAC GTG TCA CGT dTdT-3′; 2nd chain 5′- ACG UGA CAC GUU CGG AGA AUU dTdT-3′) were synthesized at the Institute of Physical Organic Chemistry of NASB (IPOC NASB) according to known methods [53,54]. Lyophilized siRNAs were dissolved in an siRNA buffer. The composition of the siRNA buffer was 60 mM KCl (Fisher Scientific UK Ltd., Loughborough, UK), 6 mM HEPES-pH 7.5 (MilliporeSigma, Burlington, MA, USA), and 0.2 mM MgCl_2_ (MilliporeSigma, Burlington, MA, USA). Non-fluorescent siRNAs were used for the cytotoxicity assay; siRNAs labeled with FAM (6-carboxyfluorescein, IPOC NASB, Minsk, Belarus) at the 5′-end of the sense sequence were used in flow cytometric experiments. Non-coding double-stranded RNA (ntRNA, non-target), which has no targets in the studied cell lines (23 bp), was used as a control. 

### 4.2. Silver Nanoparticles

**Figure 16:**
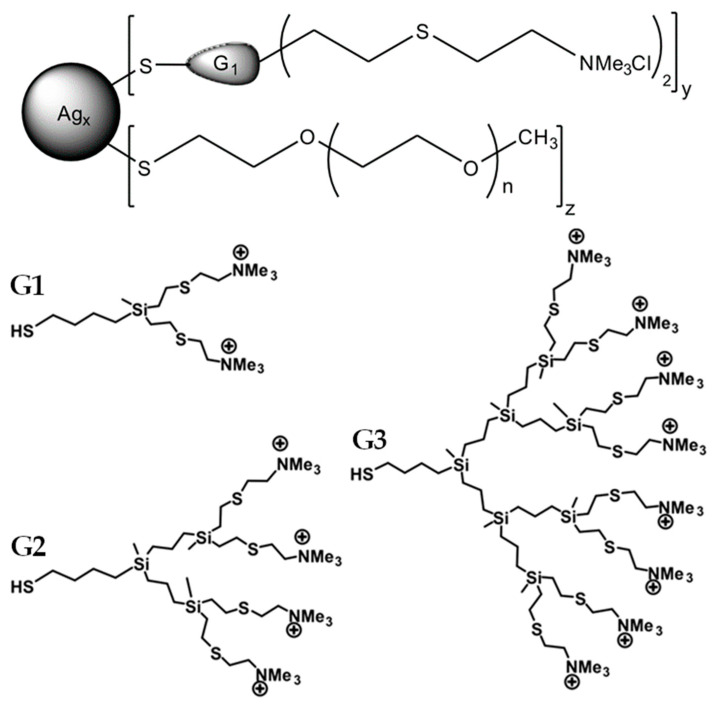
Structures of AgNPs and dendrons. The structure of silver nanoparticles is shown at the top of the figure: *y:z* is the ratio of dendrons to PEG; *n* characterizes the PEG length and is equal to 1, 2, and 3 for dendrons of the 1st, 2nd, and 3rd generation, respectively. Structural formulas of dendrons’ 1st (G1), 2nd (G2) and 3rd (G3) generations are at the bottom of the figure.

Synthesis and characteristics of dendronized silver nanoparticles with cationic carbosilane dendrons have previously been described [32,55]. In this work, silver nanoparticles were used (AgNP, Figure 16, Table 1) whose surface was modified with both dendrons and short chains of polyethylene glycol (PEG) in various ratios [34]. 

The nanoparticles were synthesized and transferred free of charge to the research team. Biofunctionalized AgNP were produced in water by the direct reaction of AgNO_3_, of the cationic dendrons with a thiol moiety at the focal point HSG_n_(S-NMe^3+^)_m_ (*n* = 1, m = 2 (G1); *n* = 2, m = 4 (G2); *n* = 3, m = 8 (G3), Figure 16), of commercial PEG ligand CH_3_O(CH_2_CH_2_O)_n_CH_2_CH_2_SH, and of NaBH_4_ acting as the reducing agent. 

### 4.3. Analysis of Complexes

#### 4.3.1. Gel-Electrophoresis

Agarose gel electrophoresis was used to analyze the formation of complexes between AgNP and FAM-labeled siRNAs (siMCL-xL, 1.5 μM) in different mass-molar ratios. Dendronized nanoparticles have a cationic charge on the surface and bind negatively charged siRNAs. The formed complexes are unable to move in the pores of the gel under a constant electric field due to their size. 

Prepared complexes were incubated for 20 min (or other period of time to determine stability) at room temperature (25 °C) in 10 mM PBS (PBS tablets, Fisher Scientific UK Ltd., Loughborough, UK). An amount of 30% glycerol was added to the samples in a 6:1 ratio as a loading buffer, after which the samples were placed into the wells. Complex formation was assessed by the retardation of migration of the siRNAs. Gel electrophoresis was performed in TAE buffer (MilliporeSigma, Burlington, MA, USA) at 40 V for 30 min. After electrophoresis, the gel was visualized using UV light and digital images of the stained gel were taken by VersaDoc™ (Bio-Rad Laboratories, Inc., Hercules, CA, USA).

#### 4.3.2. Circular Dichroism

The circular dichroism (CD) spectra of complexes based on AgNP and siRNA were measured using a Jasco J-815 CD spectrometer (Jasco, Tokyo, Japan). The siRNA concentration was 1 μM. All measurements were carried out in PBS, pH 7.4, at 25 °C. CD spectra were measured in the range from 200 to 300 nm using a quartz cell with a 5 mm optical path length. The scanning speed was 50 nm/min and the bandwidth was 1.0 nm. The measurements were carried out starting with siRNA without AgNP followed by titration with nanoparticles. After each dropping of AgNP, the samples were incubated for 5 min. The analysis took into account the peaks of the CD spectra of siRNAs at λ = 260 nm (positive peak).

#### 4.3.3. Zeta-Potential and Dynamic Light Scattering

Measurements of hydrodynamic size and zeta-potential were performed with the use of a Zetasizer Nano ZS (Malvern Instruments Ltd., Worcestershire, UK). Complexes were prepared in phosphate-buffered saline (PBS) by mixing siRNA (1 μM) with an increasing concentration of nanoparticles, placed in low-volume sizing cuvettes (ZEN0112, Malvern) for hydrodynamic size determination or in folded capillary cells (DTS 1070, Malvern) for zeta-potential measurements at 37 °C. The data were analyzed using Malvern software. Particle size distribution was determined by a multimodal peak analysis. When the polydispersity index (PDI) was lower than 0.5, Z-average was taken into account; when the PDI was higher than 0.5, individual peaks were analyzed. Data were presented as mean ± SD, *n* = 4 (15 measurements each).

### 4.4. Evaluation of Toxicity of Nanoparticles for Blood Cells

#### 4.4.1. Hemolytic Activity of Silver Nanoparticles

AgNPs prepared in PBS were added to the erythrocytes (at 2% hematocrit). The samples were incubated at 37 °C for 2 and 24 h, centrifuged (3000× *g*, 10 min, 4 °C), and their absorbance measured at 540 nm using the spectrophotometer Jasco V-630 (Jasco, Tokyo, Japan). The percentage of hemolysis was calculated using the following formula:

(1) $$H\left(\%\right)=\frac{A_{540}}{A_{water}}\times 100\%$$

where *A*_540_ is the sample absorption; *A_water_* is the positive control of RBC in water (100% release of hemoglobin).

Data were presented as percentage of hemolysis, mean ± SD, *n* = 4.

#### 4.4.2. PBMC Inhibition

PBMCs were seeded into 96-well black plates at a density of 1 × 10^5^ cells per well. The cells were treated with AgNPs in various concentrations for 72 h. Following the incubation, resazurin was added to the culture medium to a final concentration of 10 µg/mL and the plates were incubated at 37 °C in darkness to allow conversion of resazurin to resorufin. The fluorescence of metabolized resazurin in the cell suspension was measured after 90 min at 530 nm excitation and 590 nm emission using a Wallac 1420 Multilabel Counter (Wallac Oy PerkinElmer, Turku, Finland). Viability of cells (relative units—r.u.) was calculated relative to the control (not treated).

### 4.5. Cell Line Cultures

Experiments were carried out on HeLa, human acute promyelocytic leukemia (HL60), and T-cell acute lymphoblastic leukemia (CEM-SS). HeLa cells were grown in full DMEM with stable glutamine, 4.5 g/L of glucose (Life Technologies, Paisley, UK), 10% FBS (Life Technologies, Paisley, UK), 100 U/mL of penicillin, and 0.1 mg/mL of streptomycin (MilliporeSigma, Burlington, MA, USA) at 37 °C in a humidified air atmosphere with 5% CO_2_. The HL60 and CEM-SS cells were cultured in full RPMI-1640 with stable glutamine (Life Technologies, Paisley, UK), 10% FBS (Life Technologies, Paisley, UK), 100 U/mL of penicillin, and 0.1 mg/mL of streptomycin (MilliporeSigma, Burlington, MA, USA) under the same conditions as HeLa.

### 4.6. Cellular Uptake

The cell lines were seeded at 2 × 10^5^ cells/mL in 500 µL of suitable culture medium on 24-well plates (1 × 10^5^ cells/well) and preincubated for 24 h at 37 °C in humidified air atmosphere with 5% CO_2_ before treatment. Complexes of FAM-labeled siRNA and AgNP were added to the cells. Complexes for treatment were incubated for 15 min in PBS using concentrations required for the delivery of 100 nM siRNA-FAM. The cells were incubated for 3 or 24 h and washed in PBS according to the standard procedure.

Samples were analyzed by flow cytometry (CytoFLEX, Beckman Coulter, Indianapolis, IN, USA): 25,000 events with the exclusion of necrotic (by 7-AAD; the number of necrotic cells < 1%) and aggregated (SSC-H/SSC-A gate) cells.

### 4.7. Cytotoxicity Studies

The cell lines were seeded at 1 × 10^5^ cells/mL in 100 µL of suitable culture medium on 96-well plates (1 × 10^4^ cells/well). They were preincubated for 24 h and a further 72 h after adding complexes. Complexes for treatment were incubated for 15 min in PBS using concentrations needed for the delivery of 100 nM of siRNA.

Cytotoxicity was evaluated by the Alamar Blue (Invitrogen™; Life Technologies, Paisley, UK) assay for HL60 and CEM-SS or the MTT-test (Carl Roth, Karlsruhe, Germany) for HeLa. Fluorescence/absorbance measurements used a Wallac 1420 Multilabel Counter (Wallac Oy PerkinElmer, Turku, Finland) at λ_ex_ = 530 nm and λ_em_ = 590 nm for Alamar Blue and λ_abs_ = 570 nm, using a reference wavelength of 630 nm for MTT. Viability of cells (relative units—r.u.) was calculated relative to the control (not treated).

### 4.8. Apoptosis-Necrosis Test

Cells were seeded at 2 × 10^5^ cells/mL in 1500 µL of suitable culture medium on 12-well plates (3 × 10^5^ cells/well) and preincubated for 24 h before treatment. FAM-labeled siRNA and AgNP complexes were incubated for 15 min in PBS (PBS tablets, Fisher Scientific UK Ltd., Loughborough, UK) using concentrations needed for the delivery of 100 nM of siRNA-FAM and then added to the cells. The cells were incubated for 48 h and washed twice in PBS. The samples were stained with Annexin V-eFluor™ 450 and 7AAD (Annexin V Apoptosis Detection Kit eFluor™ 450; Invitrogen™; Life Technologies, Paisley, UK).

Samples were analyzed by flow cytometry (CytoFLEX, Beckman Coulter, Indianapolis, IN, USA): 50,000 events were analyzed with the exclusion of aggregated cells (SSC-H/SSC-A gate). A total of 3 independent channels (PB, FITC, PerCP) were used to assess apoptosis/necrosis both in the general population and the cells that had accumulated complexes.

## 5. Conclusions

In general, the data obtained indicated that an increase in the level of PEGylation reduces the cytotoxicity of nanoparticles against tumor cells and RBC; however, there was an increased toxicity of AgNP against PBMC. The cautious use of these nanoparticles is recommended in relation to epithelial types of cancer, as, in the case of HeLa, a noticeable proliferative activity was observed while the level of internalization was quite low. The studied nanoparticles performed well in relation to leukemia cell lines, where not only were high levels of internalization observed but also a significant decrease in viability due to cell death by apoptosis mechanisms when using proapoptotic siRNA against the antiapoptotic mutant gene of the bcl2 family.

## Figures and Tables

**Table 1:** Nomenclature of AgNP depending on the degree of PEGylation and generation of surface dendrons.

Dendron Generation	Group Name	Dendron/PEG Molar Ratio		
		3:1	1:1	1:3
G1	AgNP-G1	1aAg	1bAg	1cAg
G2	AgNP-G2	2aAg	2bAg	2cAg
G3	AgNP-G3	3aAg	3bAg	3cAg

## Data Availability

The data presented in this study are available on request from the corresponding author without any restrictions.

## Supplementary Materials

The following supporting information can be downloaded at: https://www.mdpi.com/article/10.3390/ijms24010840/s1.
