# Supplementary material for: Silver Nanoparticles Modified by Carbosilane Dendrons and PEG as Delivery Vectors of Small Interfering RNA
Source: Int J Mol Sci. 2023 Jan 3;24(1):840. doi: 10.3390/ijms24010840 (PMC9820844; doi:10.3390/ijms24010840)
Supplement: Supplementary file 1 [file ijms-24-00840-s001.zip › ijms-2115950-supplementary.pdf]

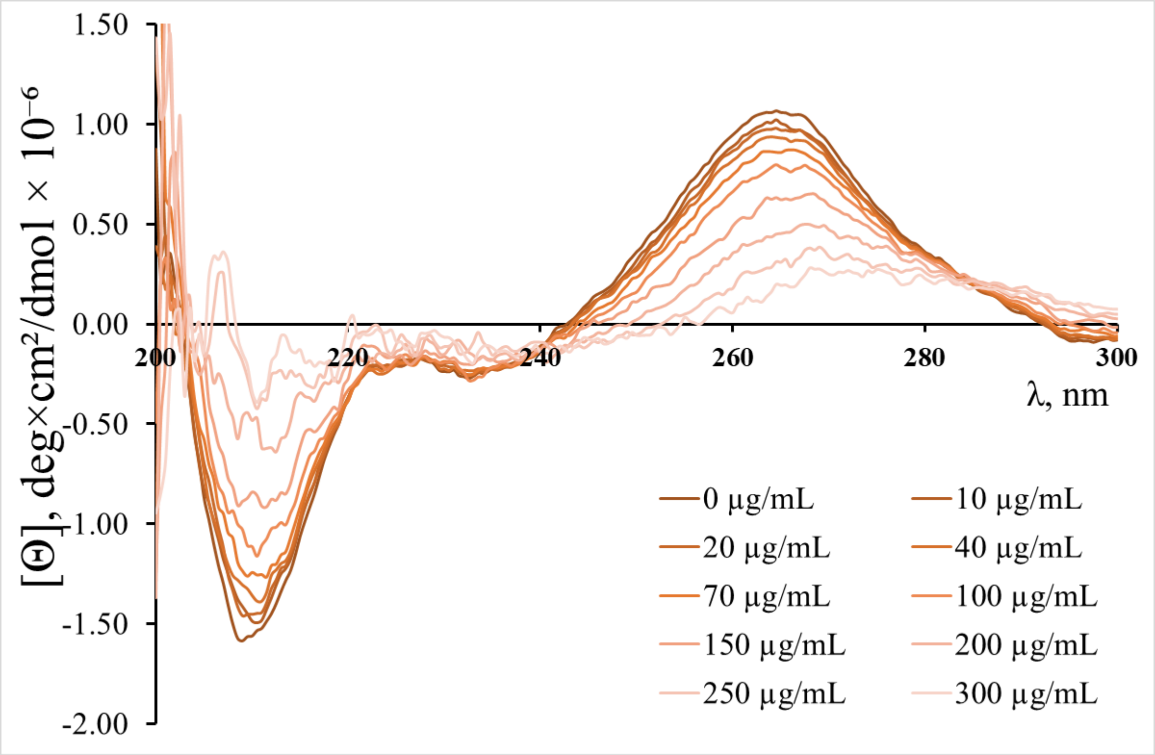

Figure S1. Circular dichroism spectra of the siRNA (siMCL-1) in the presence of 1aAg. siRNA concentration 1.5 μM; PBS, 10 mM, pH 7.4; T = 25 °C. Results represent mean obtained from a minimum of 3 independent experiments.

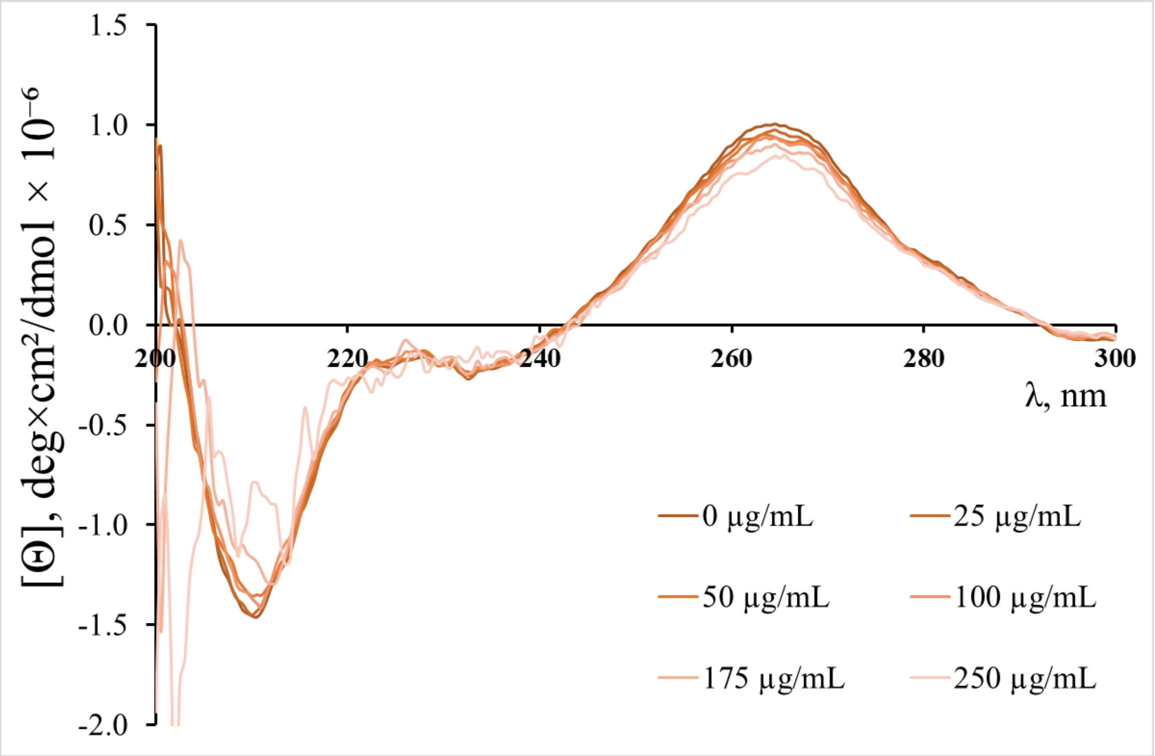

Figure S2. Circular dichroism spectra of the siRNA (siMCL-1) in the presence of 1bAg. siRNA concentration 1.5 μM; PBS, 10 mM, pH 7.4; T = 25 °C. Results represent mean obtained from a minimum of 3 independent experiments.

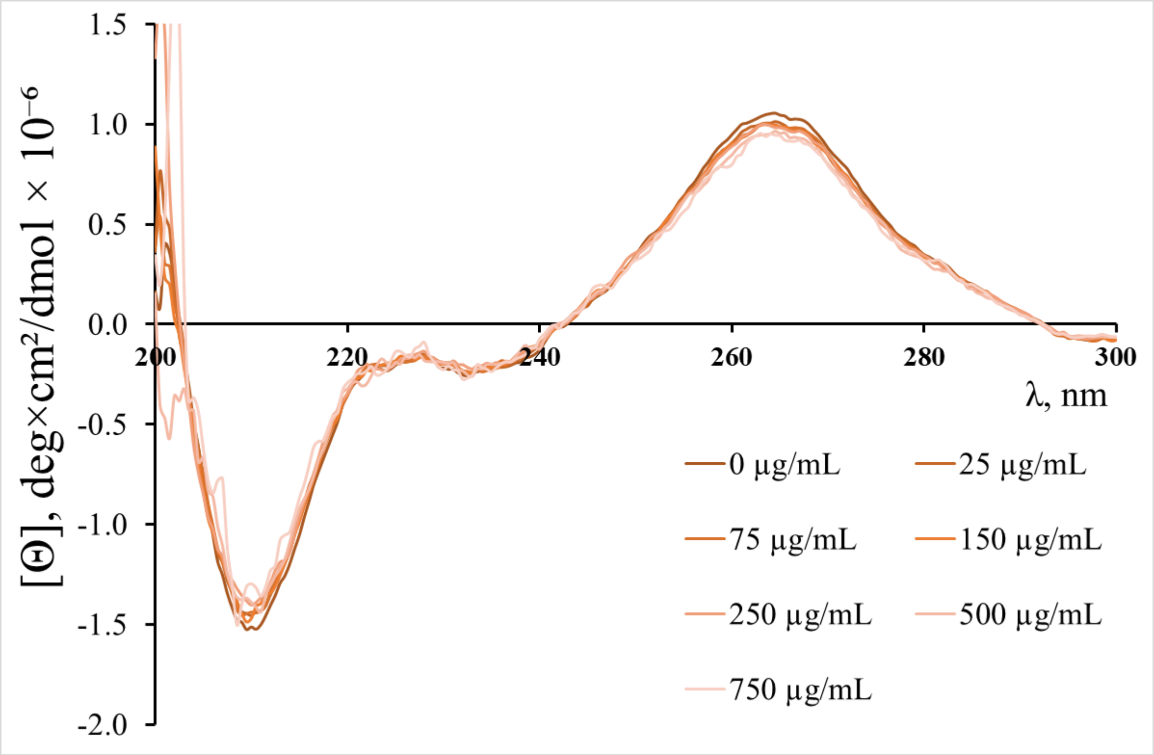

Figure S3. Circular dichroism spectra of the siRNA (siMCL-1) in the presence of 1cAg. siRNA concentration 1.5 μM; PBS, 10 mM, pH 7.4; T = 25 °C. Results represent mean obtained from a minimum of 3 independent experiments.

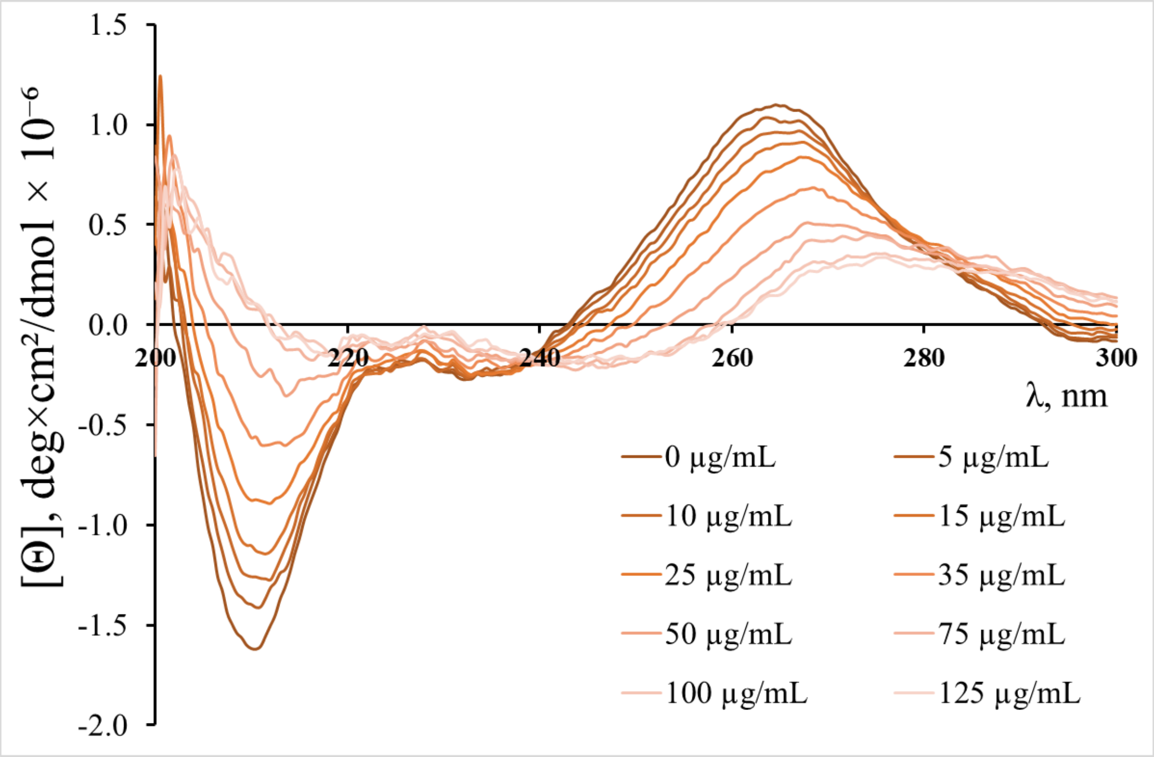

Figure S4. Circular dichroism spectra of the siRNA (siMCL-1) in the presence of 2aAg. siRNA concentration 1.5 μM; PBS, 10 mM, pH 7.4; T = 25 °C. Results represent mean obtained from a minimum of 3 independent experiments.

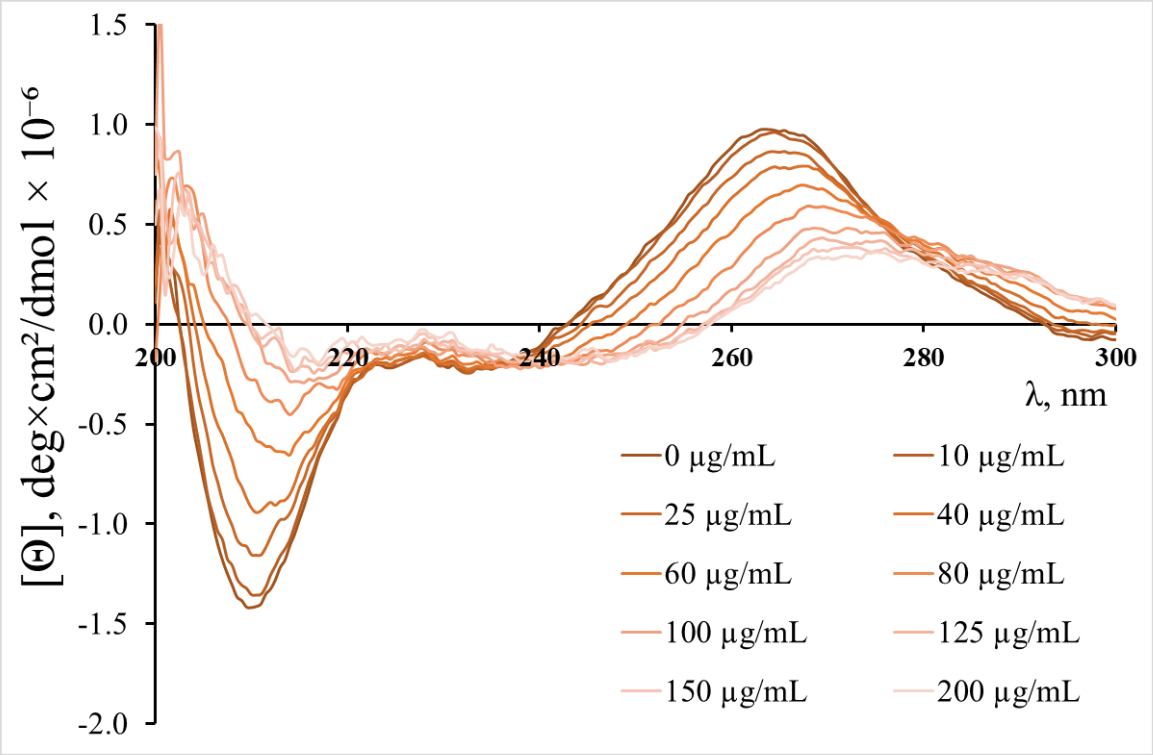

Figure S5. Circular dichroism spectra of the siRNA (siMCL-1) in the presence of 2bAg. siRNA concentration 1.5 μM; PBS, 10 mM, pH 7.4; T = 25 °C. Results represent mean obtained from a minimum of 3 independent experiments.

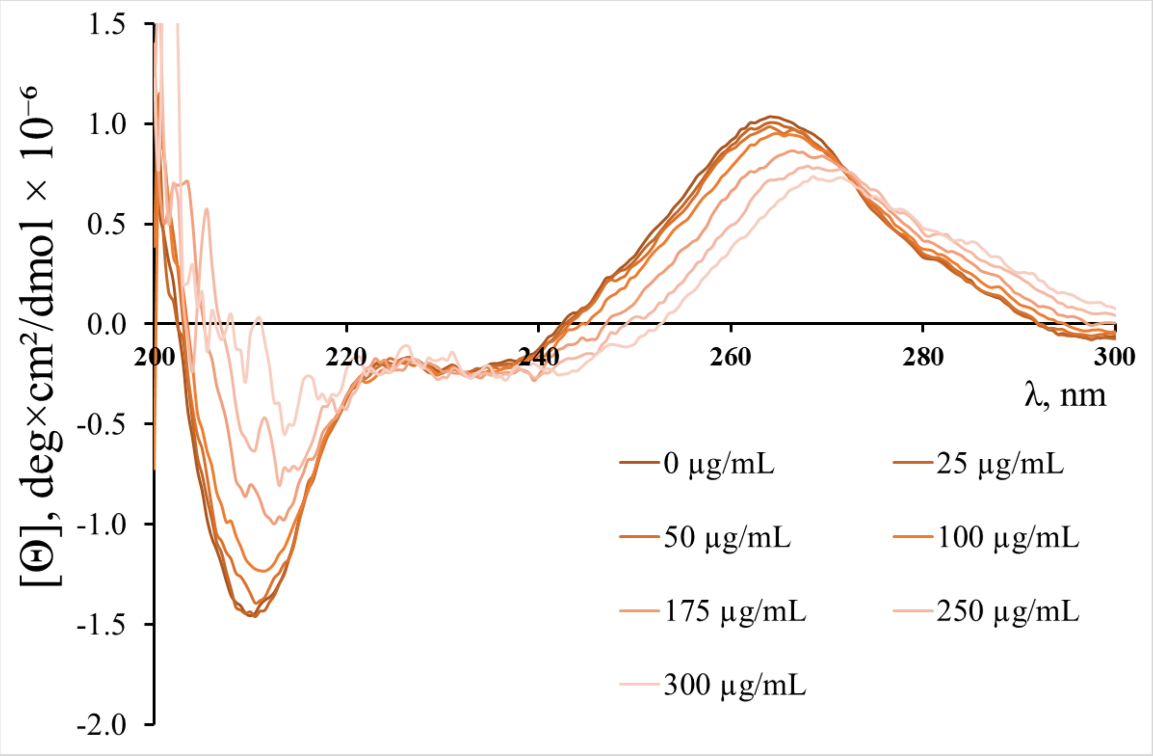

Figure S6. Circular dichroism spectra of the siRNA (siMCL-1) in the presence of 2cAg. siRNA concentration 1.5 μM; PBS, 10 mM, pH 7.4; T = 25 °C. Results represent mean obtained from a minimum of 3 independent experiments.

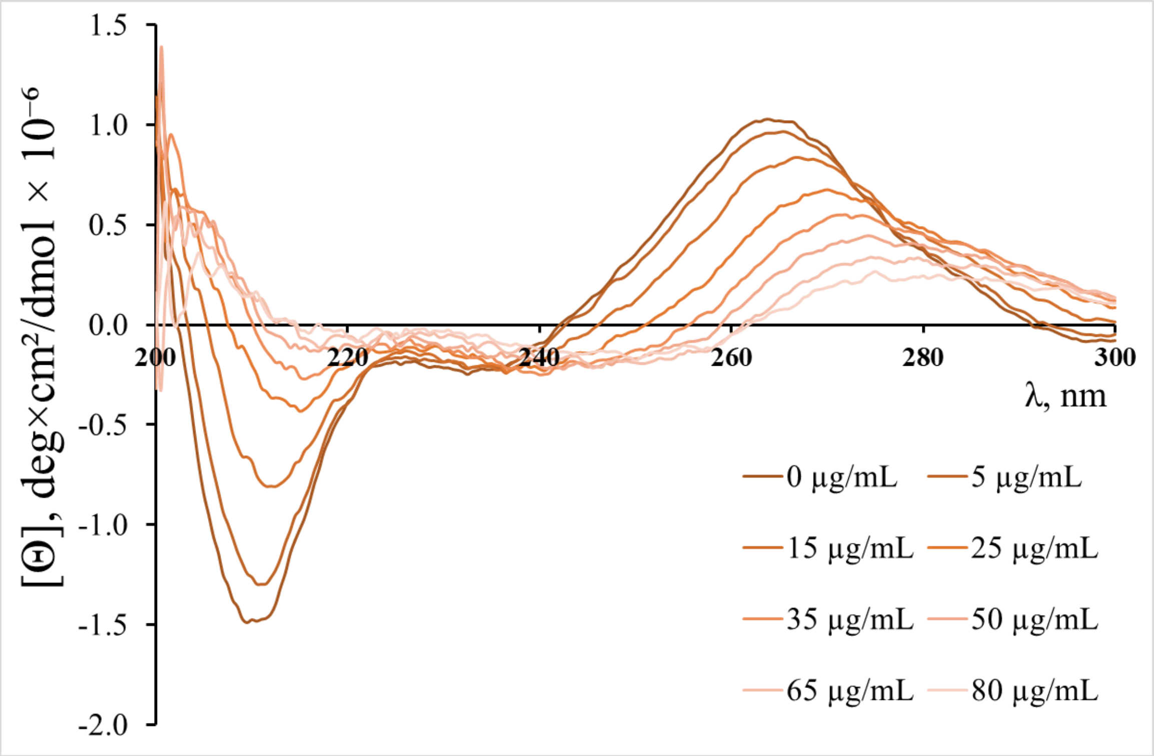

Figure S7. Circular dichroism spectra of the siRNA (siMCL-1) in the presence of 3aAg. siRNA concentration 1.5 µM; PBS, 10 mM, pH 7.4; T = 25 °C. Results represent mean obtained from a minimum of 3 independent experiments.

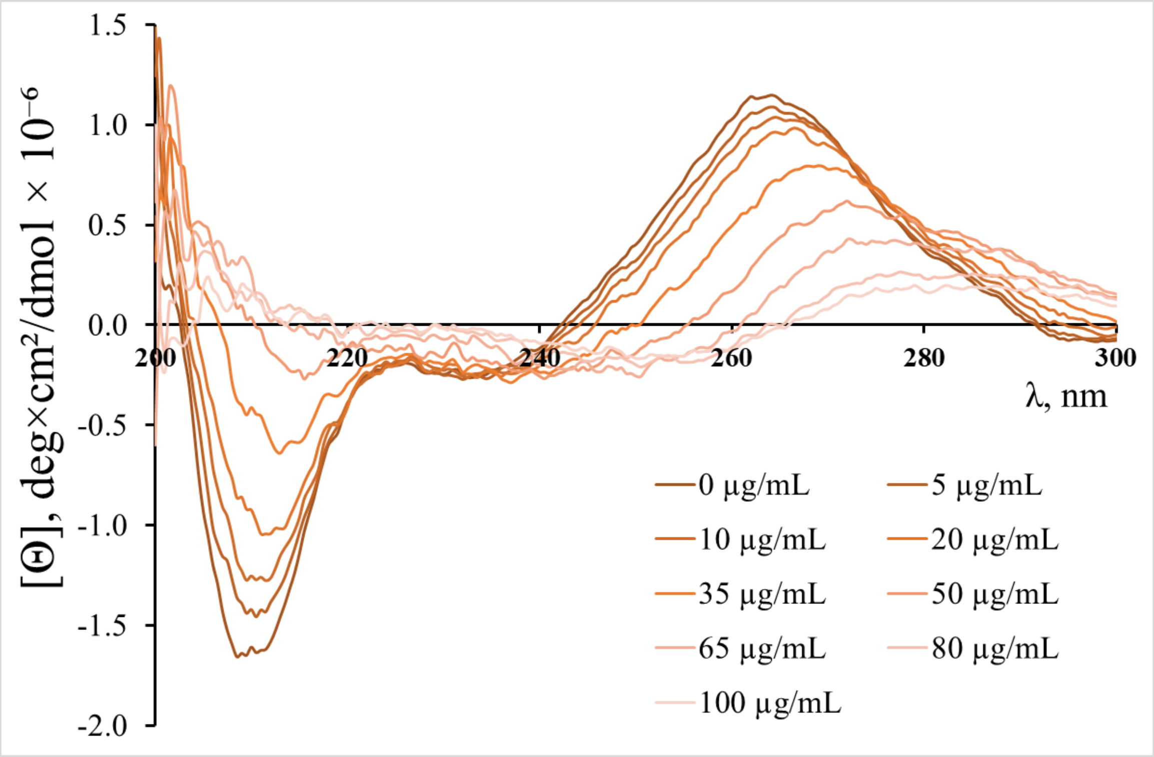

Figure S8. Circular dichroism spectra of the siRNA (siMCL-1) in the presence of 3bAg. siRNA concentration 1.5 µM; PBS, 10 mM, pH 7.4; T = 25 °C. Results represent mean obtained from a minimum of 3 independent experiments.

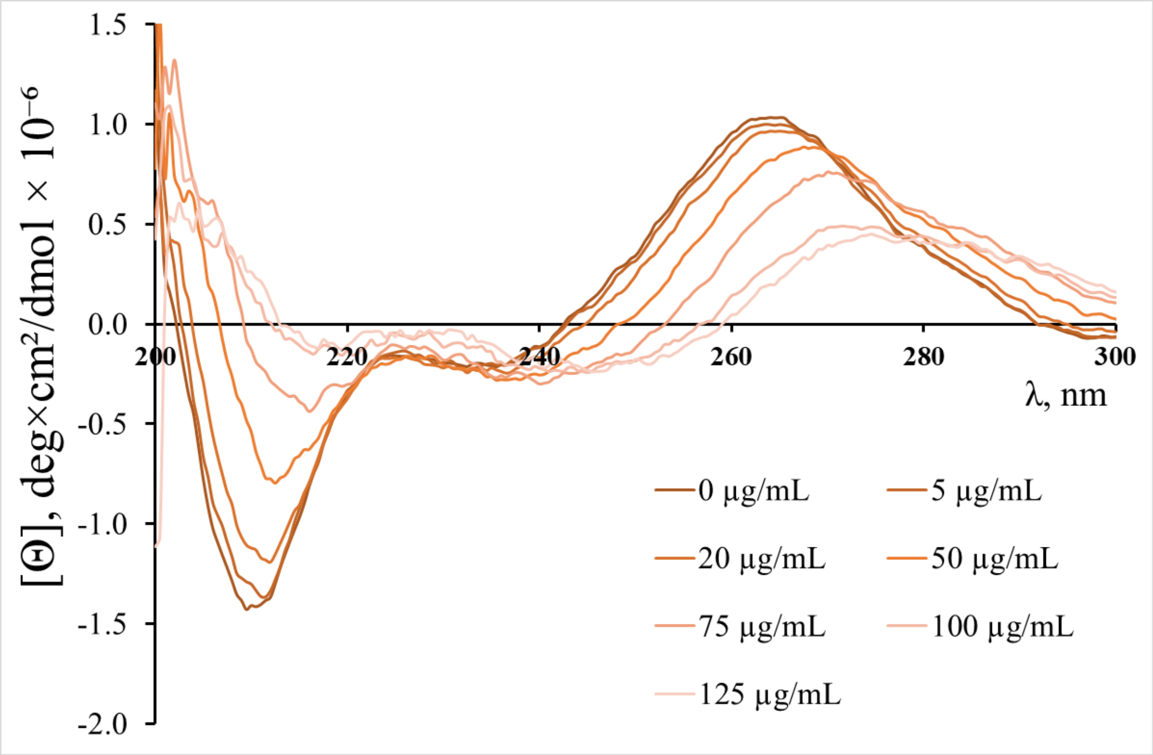

Figure S9. Circular dichroism spectra of the siRNA (siMCL-1) in the presence of 3cAg. siRNA concentration 1.5 μM; PBS, 10 mM, pH 7.4; T = 25 °C. Results represent mean obtained from a minimum of 3 independent experiments.

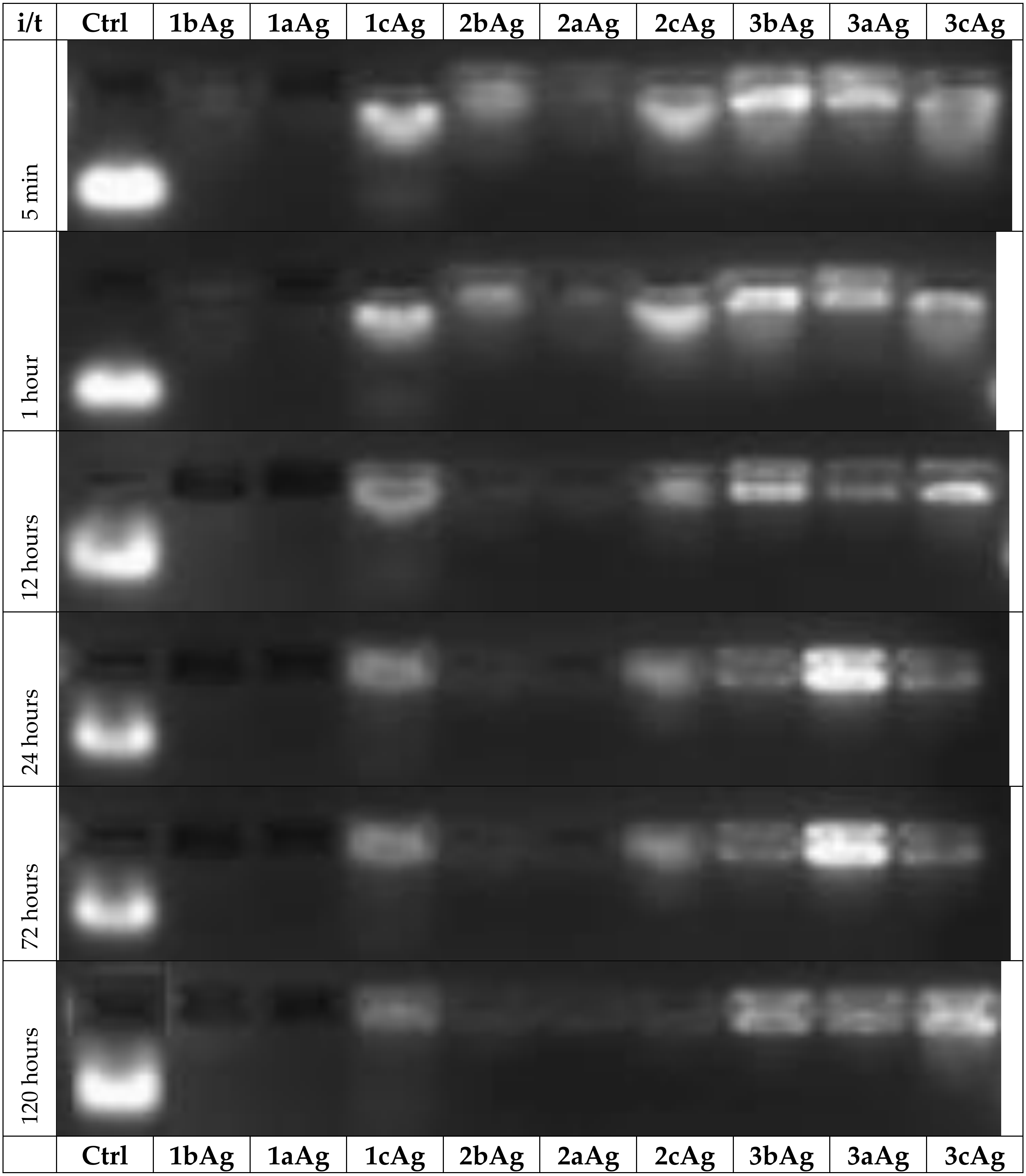

Figure S10. Gel electrophoresis of AgNP-siRNA complexes with different incubation times. The concentration of siRNA (siMCL-1) = 1.5 μM. The AgNP concentration was taken from considerations of complete siRNA binding (or close to it). The concentration of AgNP with the 1st generation of dendrons was 800 μg/mL, 2nd – 400 μg/mL, and 3rd – 200 μg/mL; T = 25 °C. “Ctrl” is non-treated siRNA control well for signal level comparison.

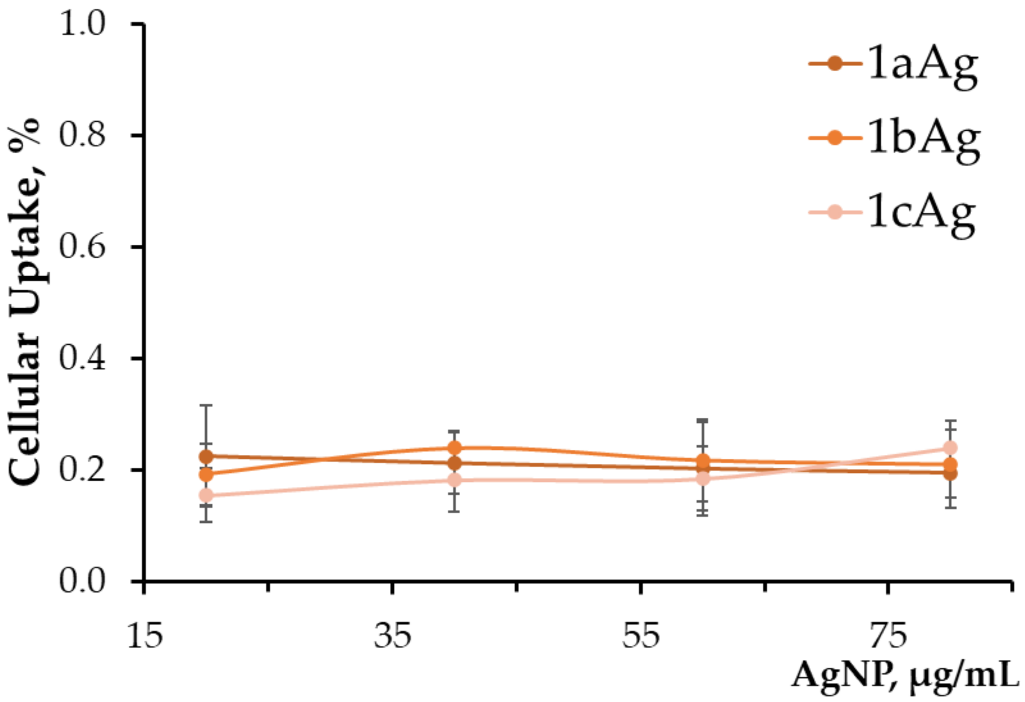

Figure S11. Cellular uptake of complexes with AgNP-G1 and siRNA (ntRNA-FAM, 100 nM) in HeLa cells after 3 h incubation. Data obtained based on fluorescence intensity from FAM-labeled RNA by flow cytometry.

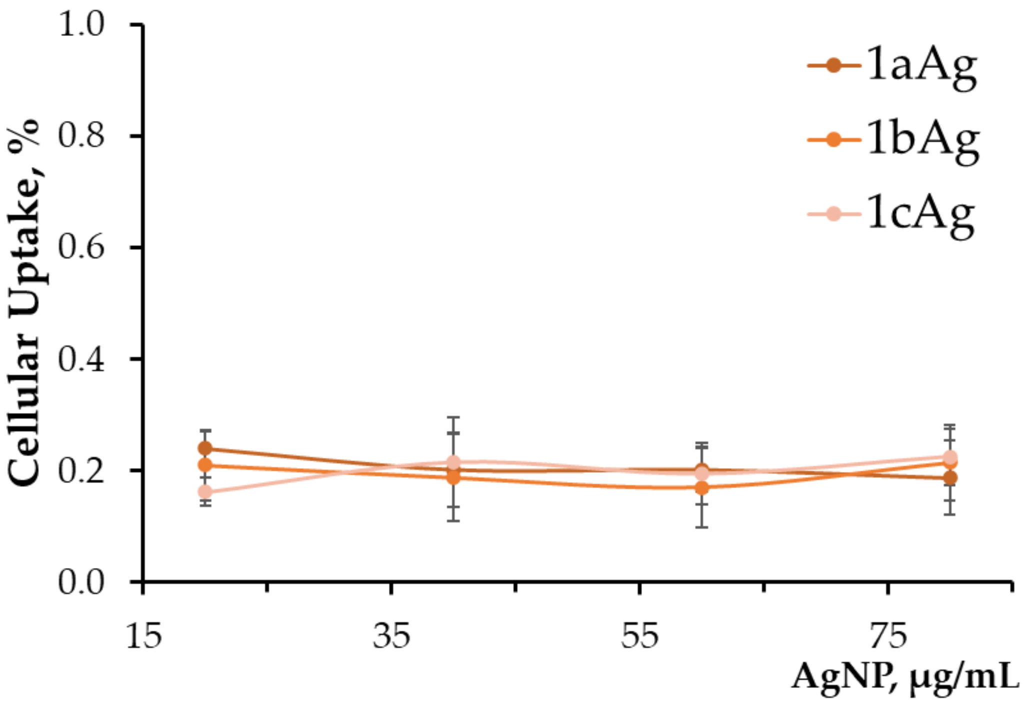

Figure S12. Cellular uptake of complexes with AgNP-G1 and siRNA (ntRNA-FAM, 100 nM) in HeLa cells after 24 h incubation. Data obtained based on fluorescence intensity from FAM-labeled RNA by flow cytometry.

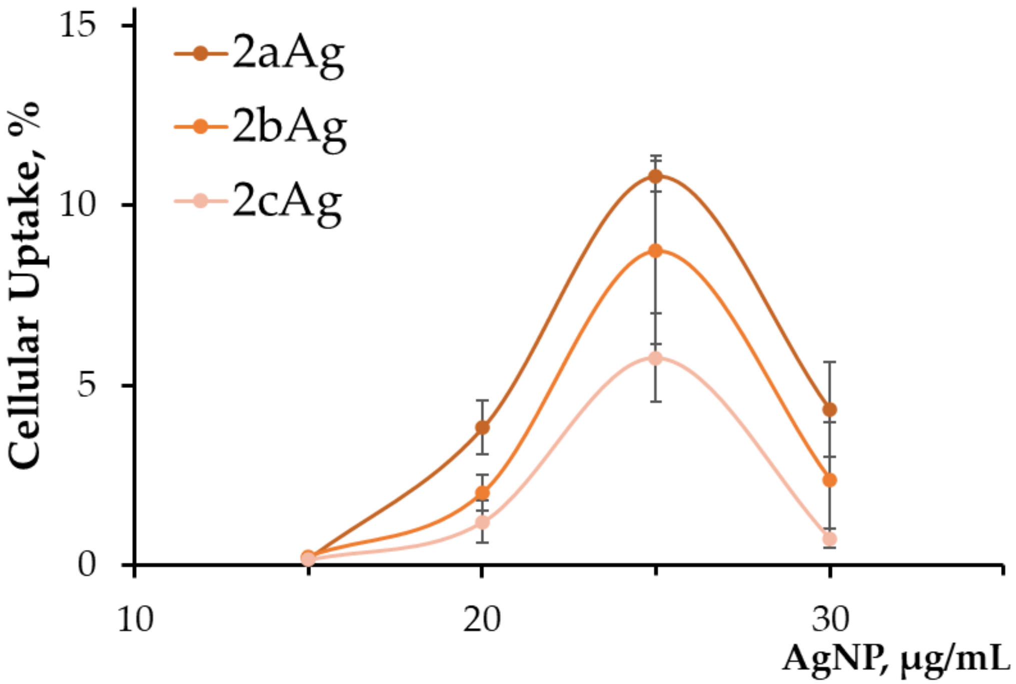

Figure S13. Cellular uptake of complexes with AgNP-G2 and siRNA (ntRNA-FAM, 100 nM) in HeLa cells after 3 h incubation. Data obtained based on fluorescence intensity from FAM-labeled RNA by flow cytometry.

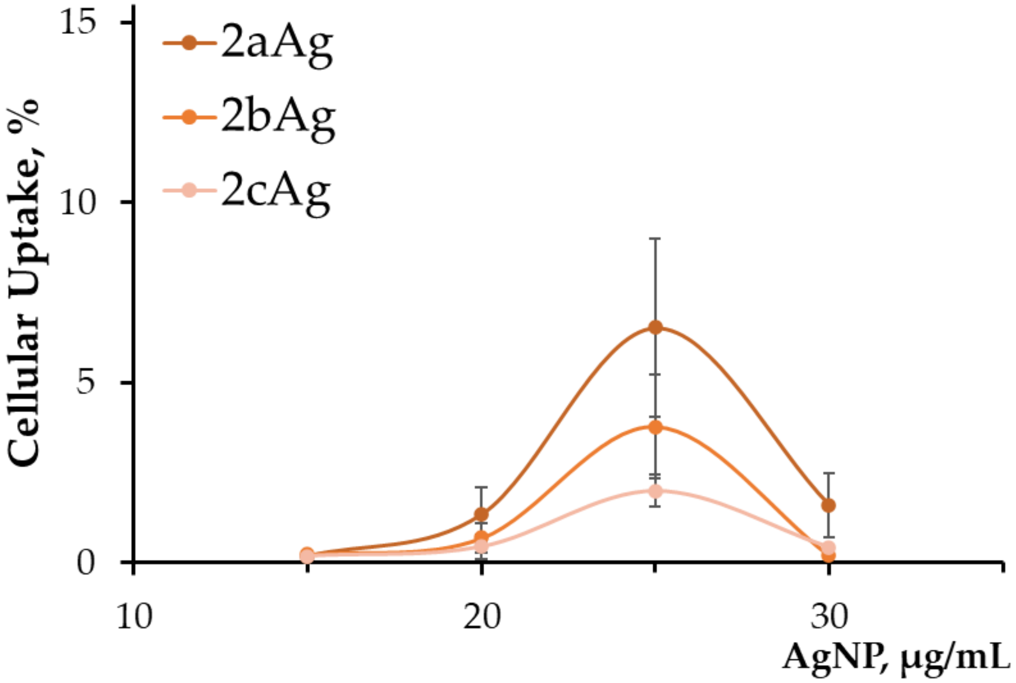

Figure S14. Cellular uptake of complexes with AgNP-G2 and siRNA (ntRNA-FAM, 100 nM) in HeLa cells after 24 h incubation. Data obtained based on fluorescence intensity from FAM-labeled RNA by flow cytometry.

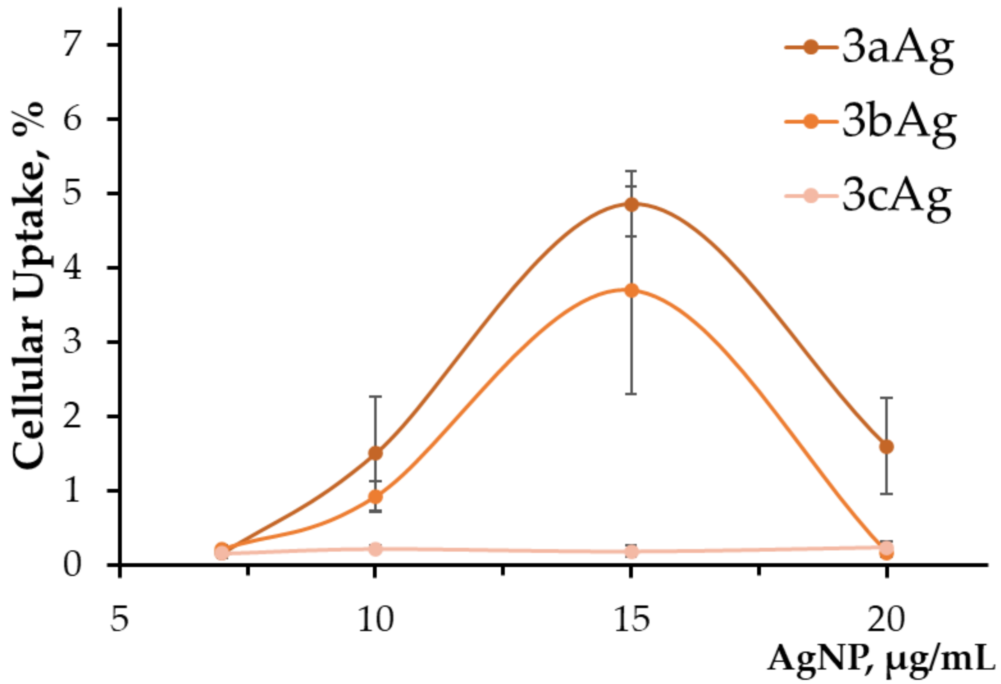

Figure S15. Cellular uptake of complexes with AgNP-G3 and siRNA (ntRNA-FAM, 100 nM) in HeLa cells after 3 h incubation. Data obtained based on fluorescence intensity from FAM-labeled RNA by flow cytometry.

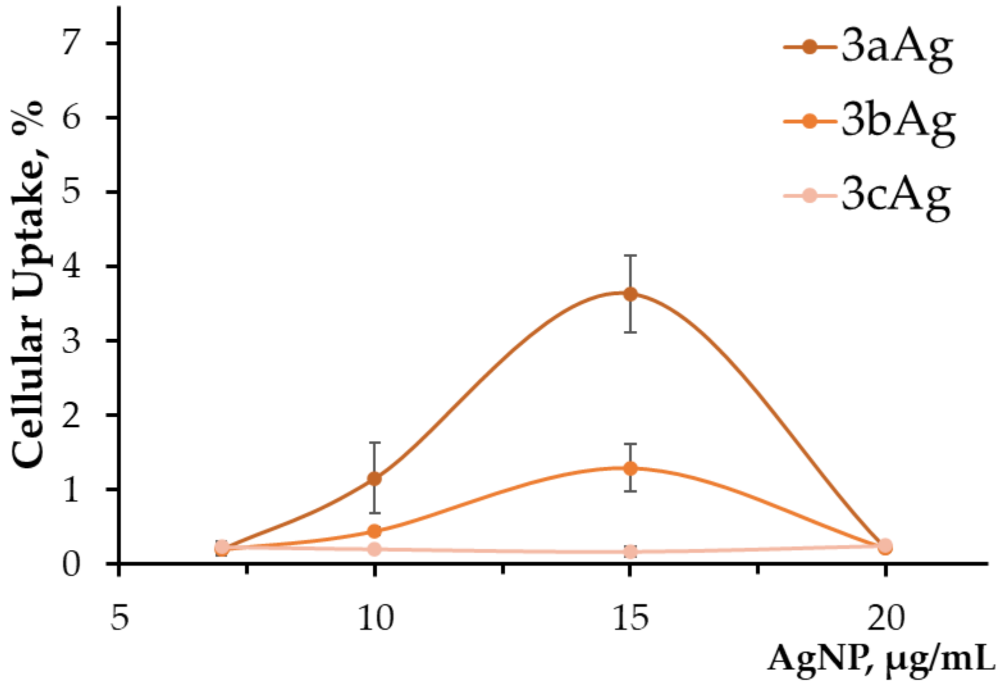

Figure S16. Cellular uptake of complexes with AgNP-G3 and siRNA (ntRNA-FAM, 100 nM) in HeLa cells after 24 h incubation. Data obtained based on fluorescence intensity from FAM-labeled RNA by flow cytometry.

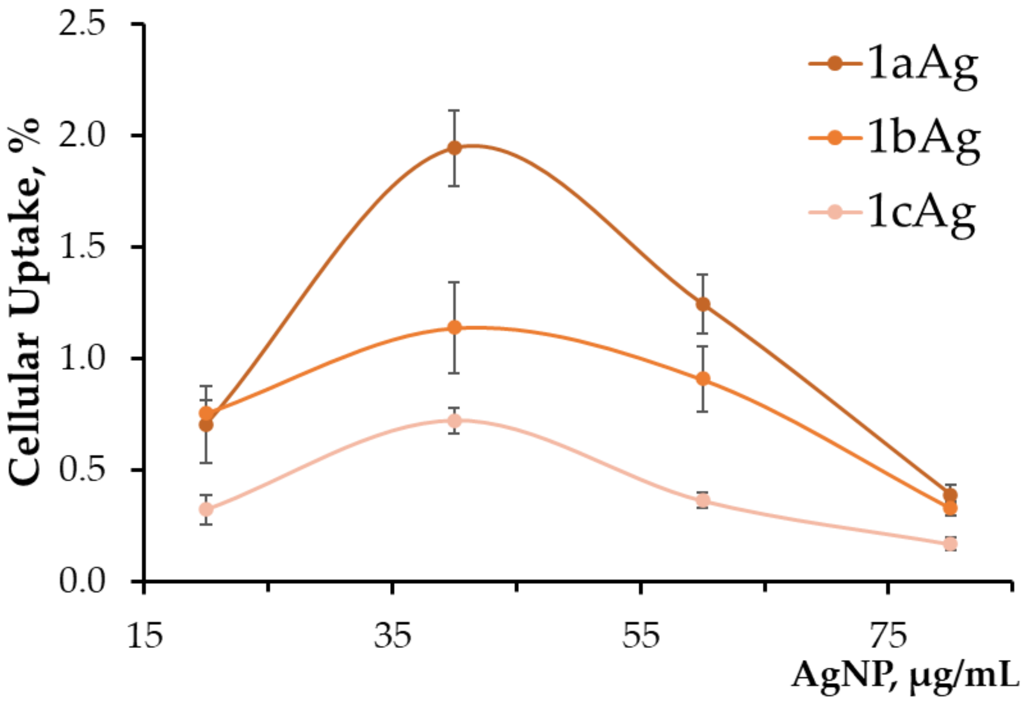

Figure S17. Cellular uptake of complexes with AgNP-G1 and siRNA (ntRNA-FAM, 100 nM) in HL60 cells after 3 h incubation. Data obtained based on fluorescence intensity from FAM-labeled RNA by flow cytometry.

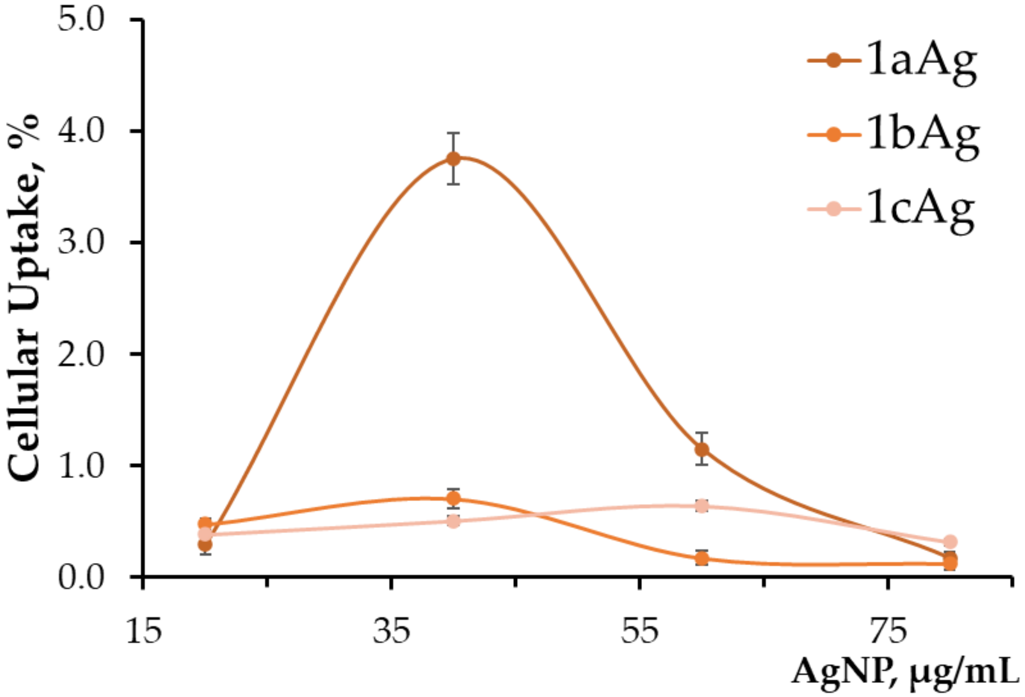

Figure S18. Cellular uptake of complexes with AgNP-G1 and siRNA (ntRNA-FAM, 100 nM) in HL60 cells after 24 h incubation. Data obtained based on fluorescence intensity from FAM-labeled RNA by flow cytometry.

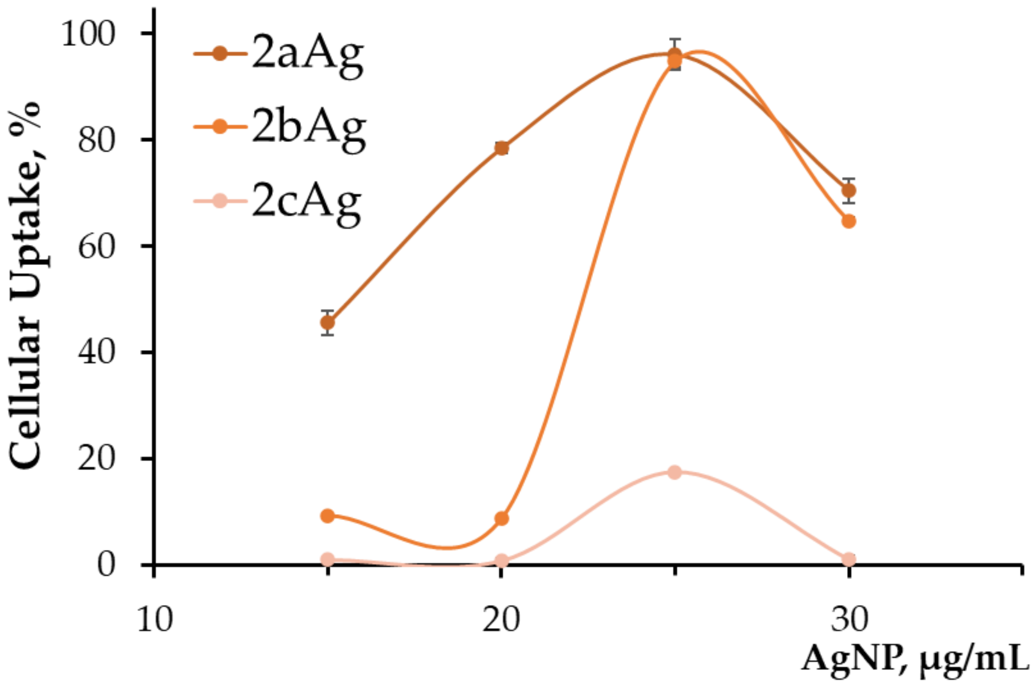

Figure S19. Cellular uptake of complexes with AgNP-G2 and siRNA (ntRNA-FAM, 100 nM) in HL60 cells after 3 h incubation. Data obtained based on fluorescence intensity from FAM-labeled RNA by flow cytometry.

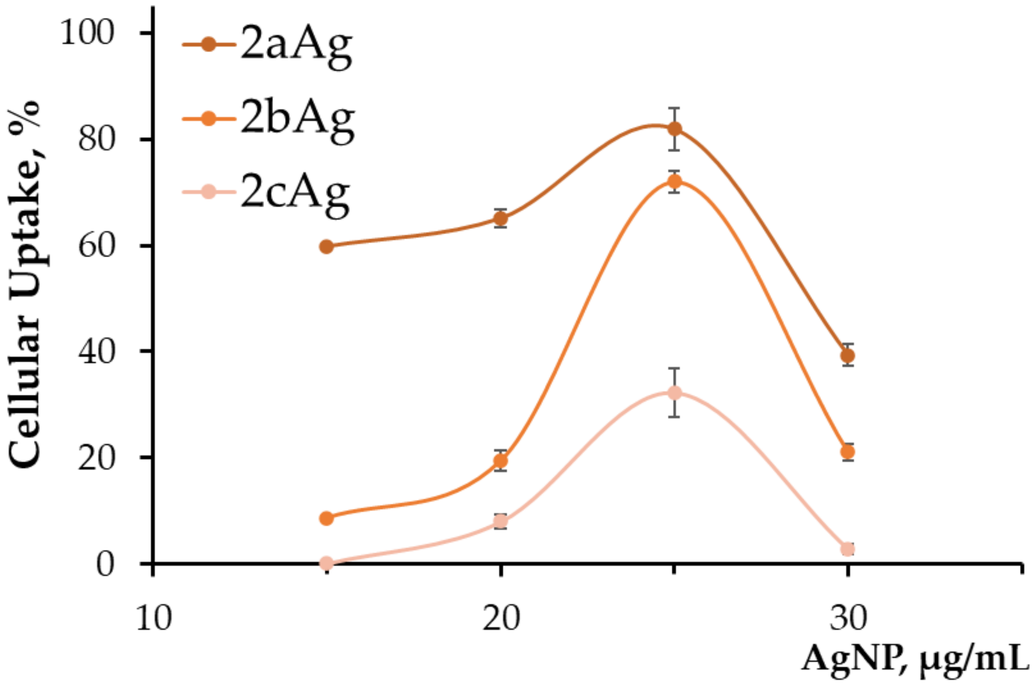

Figure S20. Cellular uptake of complexes with AgNP-G2 and siRNA (ntRNA-FAM, 100 nM) in HL60 cells after 24 h incubation. Data obtained based on fluorescence intensity from FAM-labeled RNA by flow cytometry.

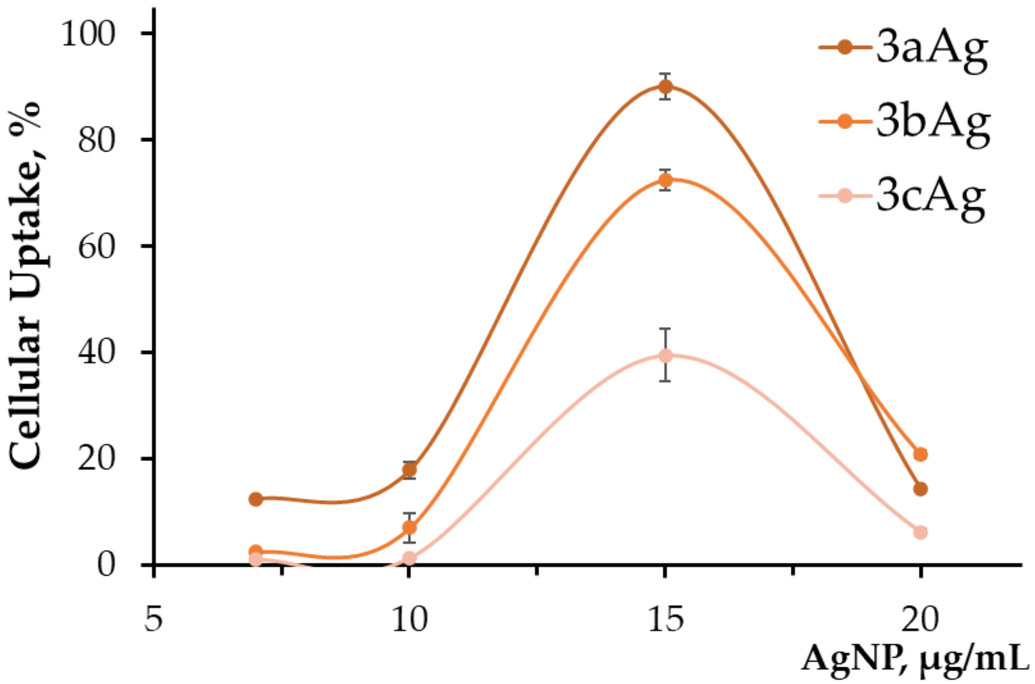

Figure S21. Cellular uptake of complexes with AgNP-G3 and siRNA (ntRNA-FAM, 100 nM) in HL60 cells after 3 h incubation. Data obtained based on fluorescence intensity from FAM-labeled RNA by flow cytometry.

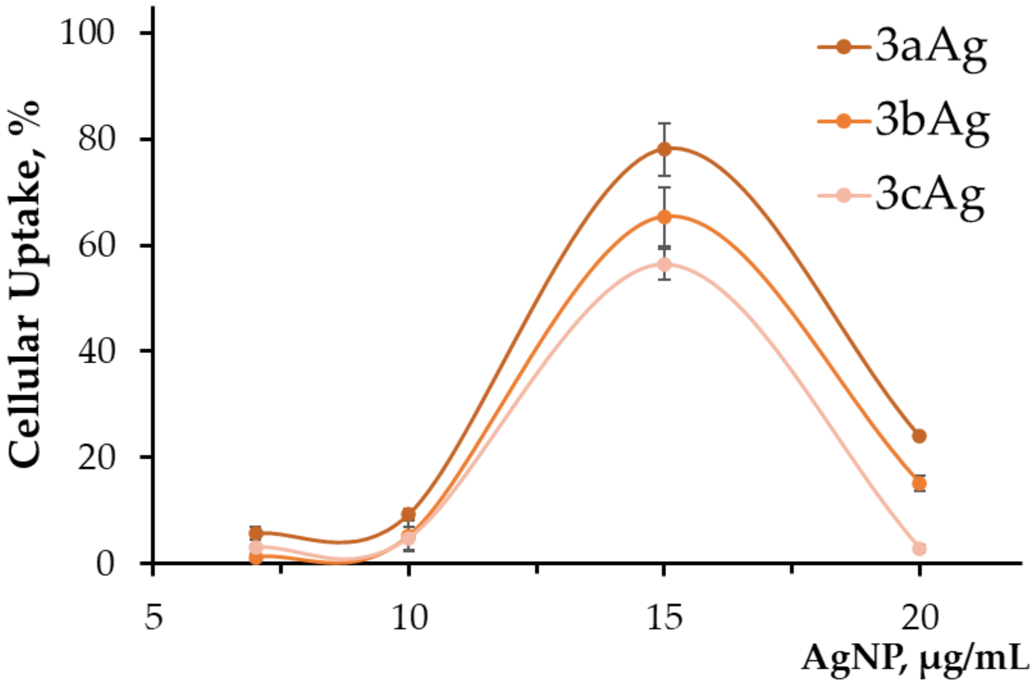

Figure S22. Cellular uptake of complexes with AgNP-G3 and siRNA (ntRNA-FAM, 100 nM) in HL60 cells after 24 h incubation. Data obtained based on fluorescence intensity from FAM-labeled RNA by flow cytometry.

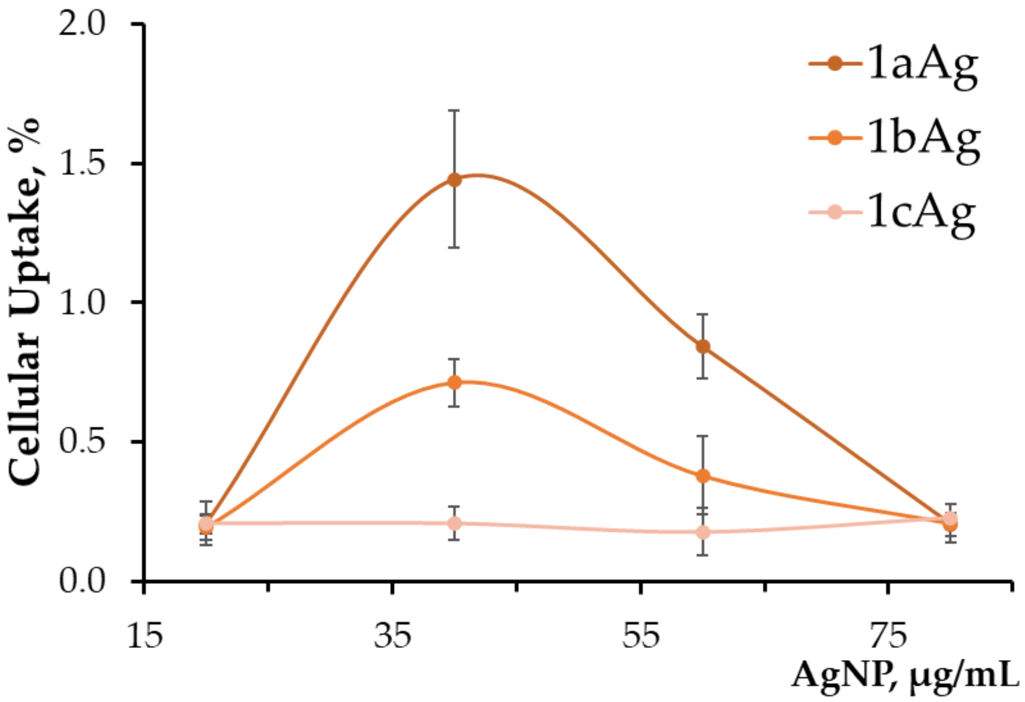

Figure S23. Cellular uptake of complexes with AgNP-G1 and siRNA (ntRNA-FAM, 100 nM) in CEM-SS cells after 3 h incubation. Data obtained based on fluorescence intensity from FAM-labeled RNA by flow cytometry.

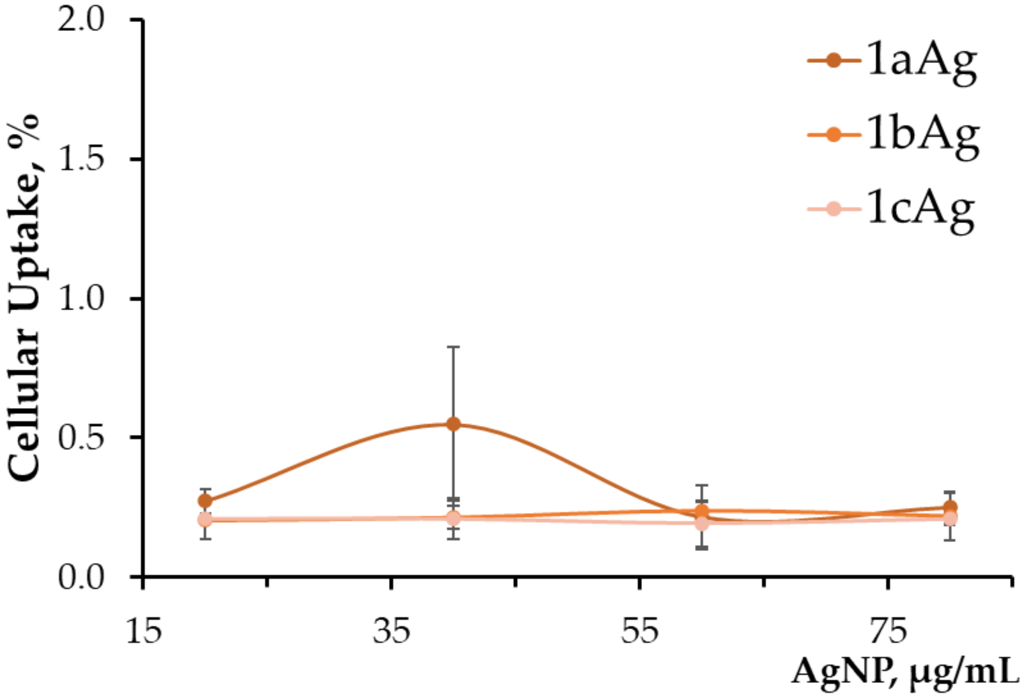

Figure S24. Cellular uptake of complexes with AgNP-G1 and siRNA (ntRNA-FAM, 100 nM) in CEM-SS cells after 24 h incubation. Data obtained based on fluorescence intensity from FAM-labeled RNA by flow cytometry.

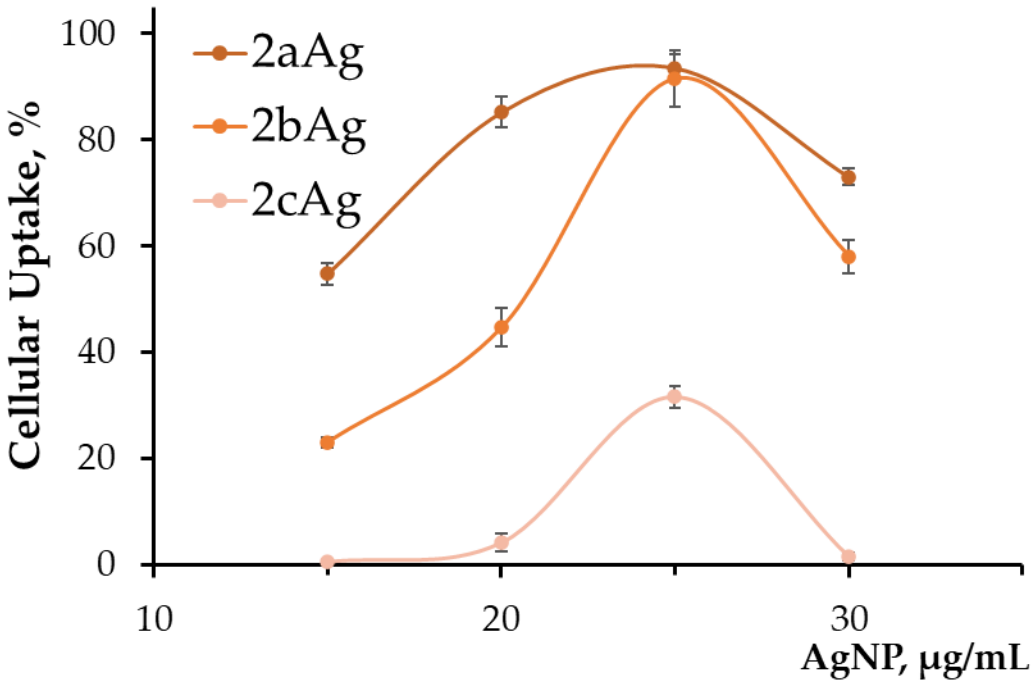

Figure S25. Cellular uptake of complexes with AgNP-G2 and siRNA (ntRNA-FAM, 100 nM) in CEM-SS cells after 3 h incubation. Data obtained based on fluorescence intensity from FAM-labeled RNA by flow cytometry.

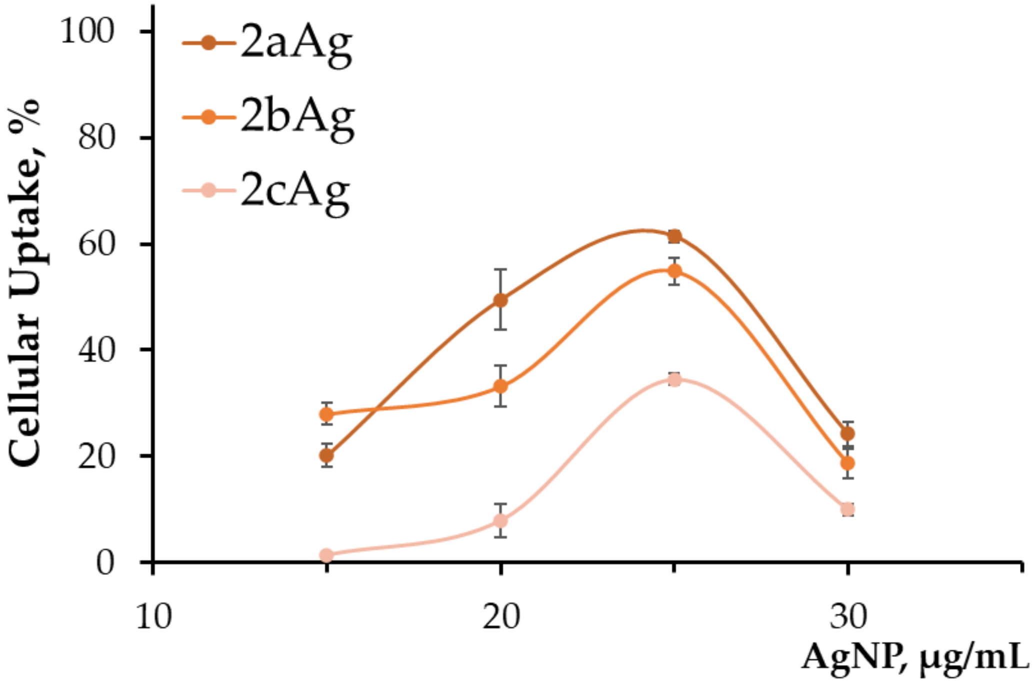

Figure S26. Cellular uptake of complexes with AgNP-G2 and siRNA (ntRNA-FAM, 100 nM) in CEM-SS cells after 24 h incubation. Data obtained based on fluorescence intensity from FAM-labeled RNA by flow cytometry.

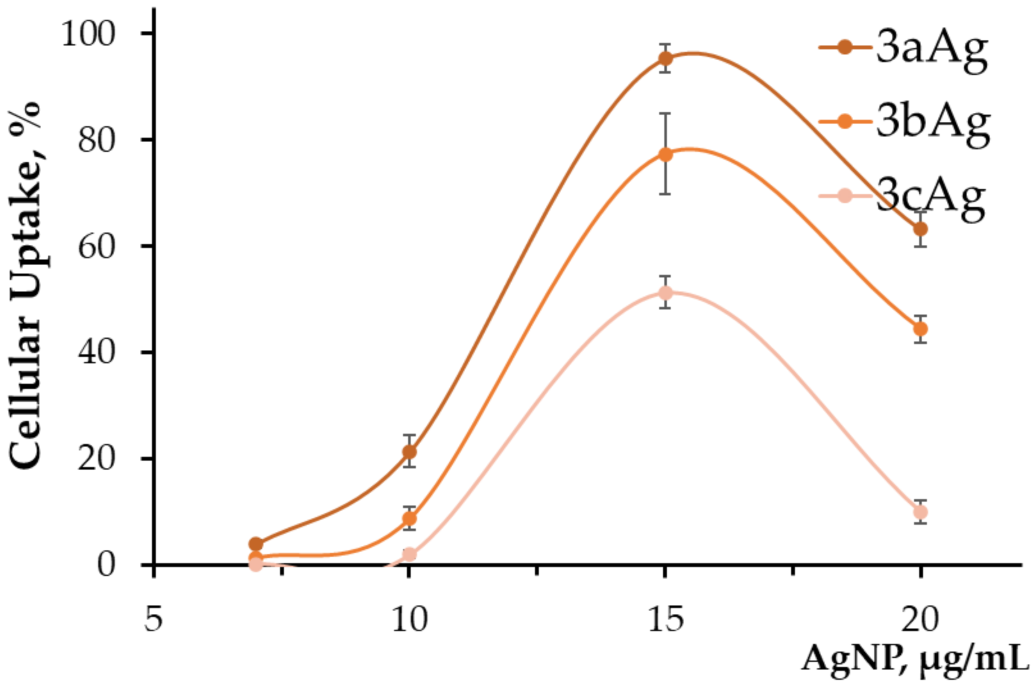

Figure S27. Cellular uptake of complexes with AgNP-G3 and siRNA (ntRNA-FAM, 100 nM) in CEM-SS cells after 3 h incubation. Data obtained based on fluorescence intensity from FAM-labeled RNA by flow cytometry.

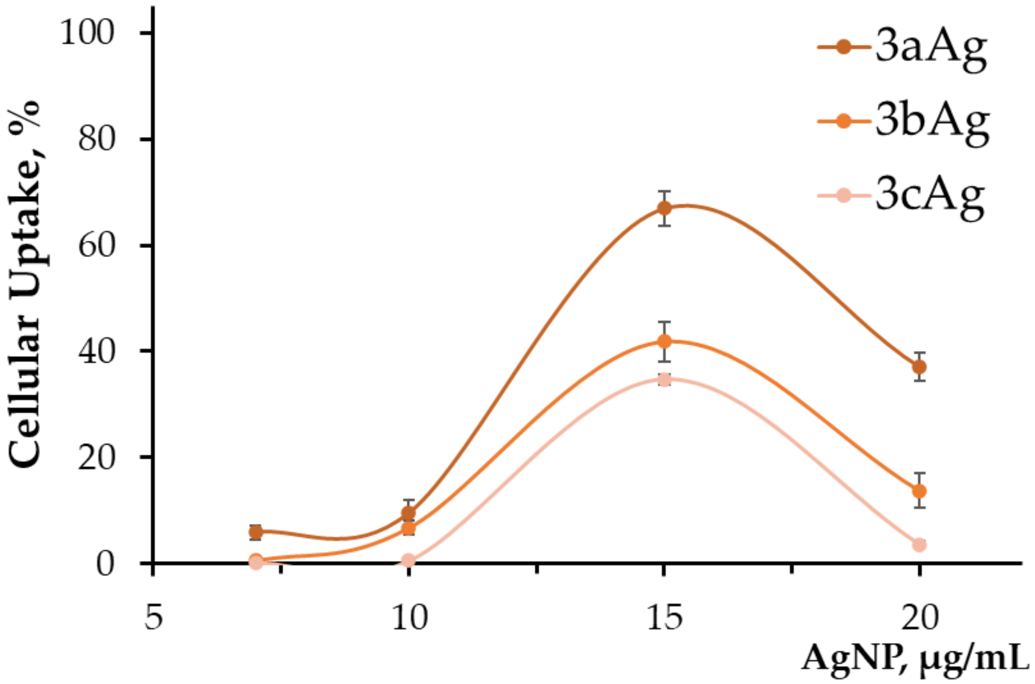

Figure S28. Cellular uptake of complexes with AgNP-G3 and siRNA (ntRNA-FAM, 100 nM) in CEM-SS cells after 24 h incubation. Data obtained based on fluorescence intensity from FAM-labeled RNA by flow cytometry.

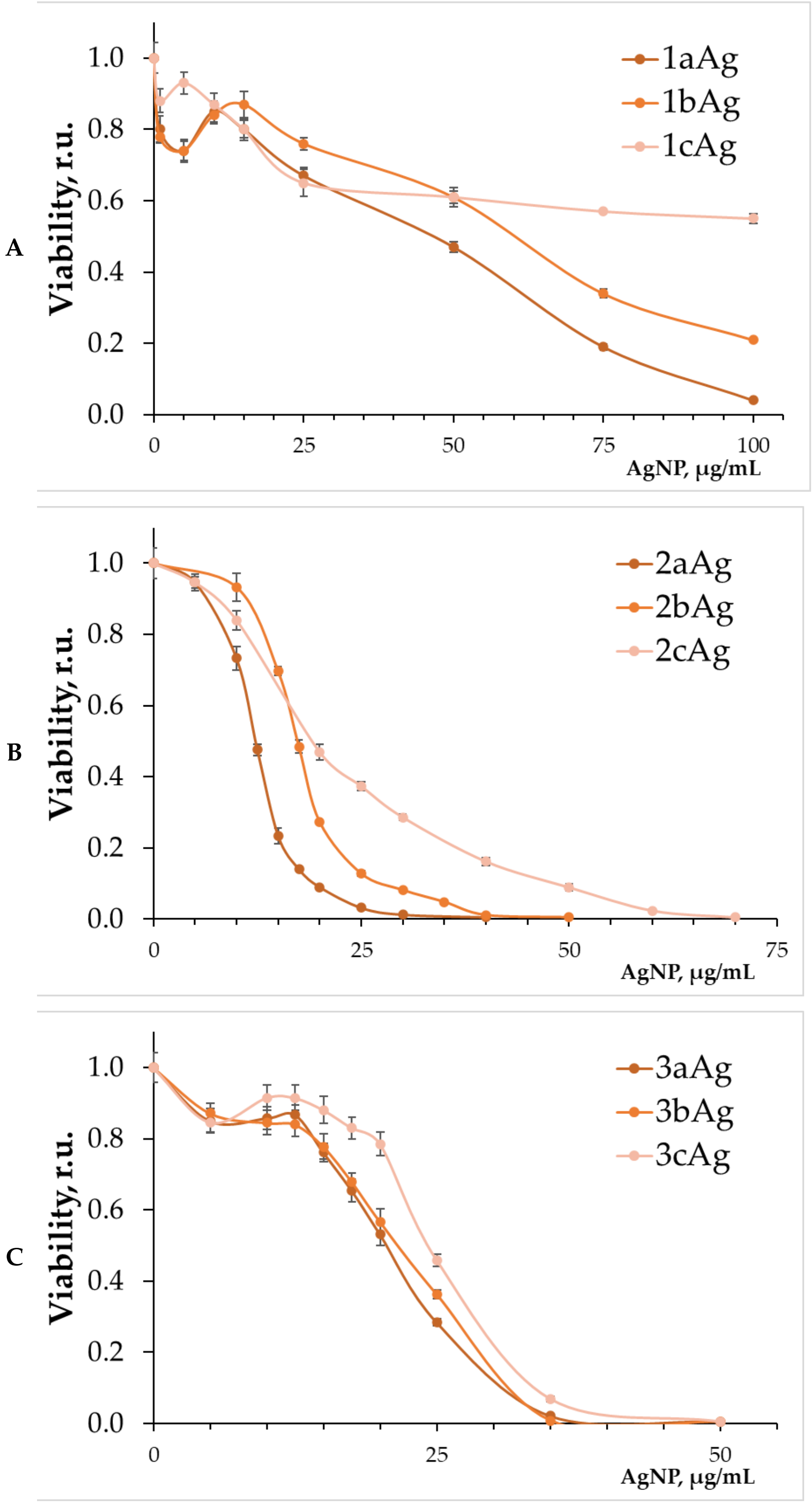

Figure S29. Dose-response curves of HL60 cells after 72 h incubation with AuNP *per se* with (A) 1st generation; (B) 2nd generation; (C) 3rd generation surface dendrons. Data obtained from MTT assay normalized to control (untreated) cells.

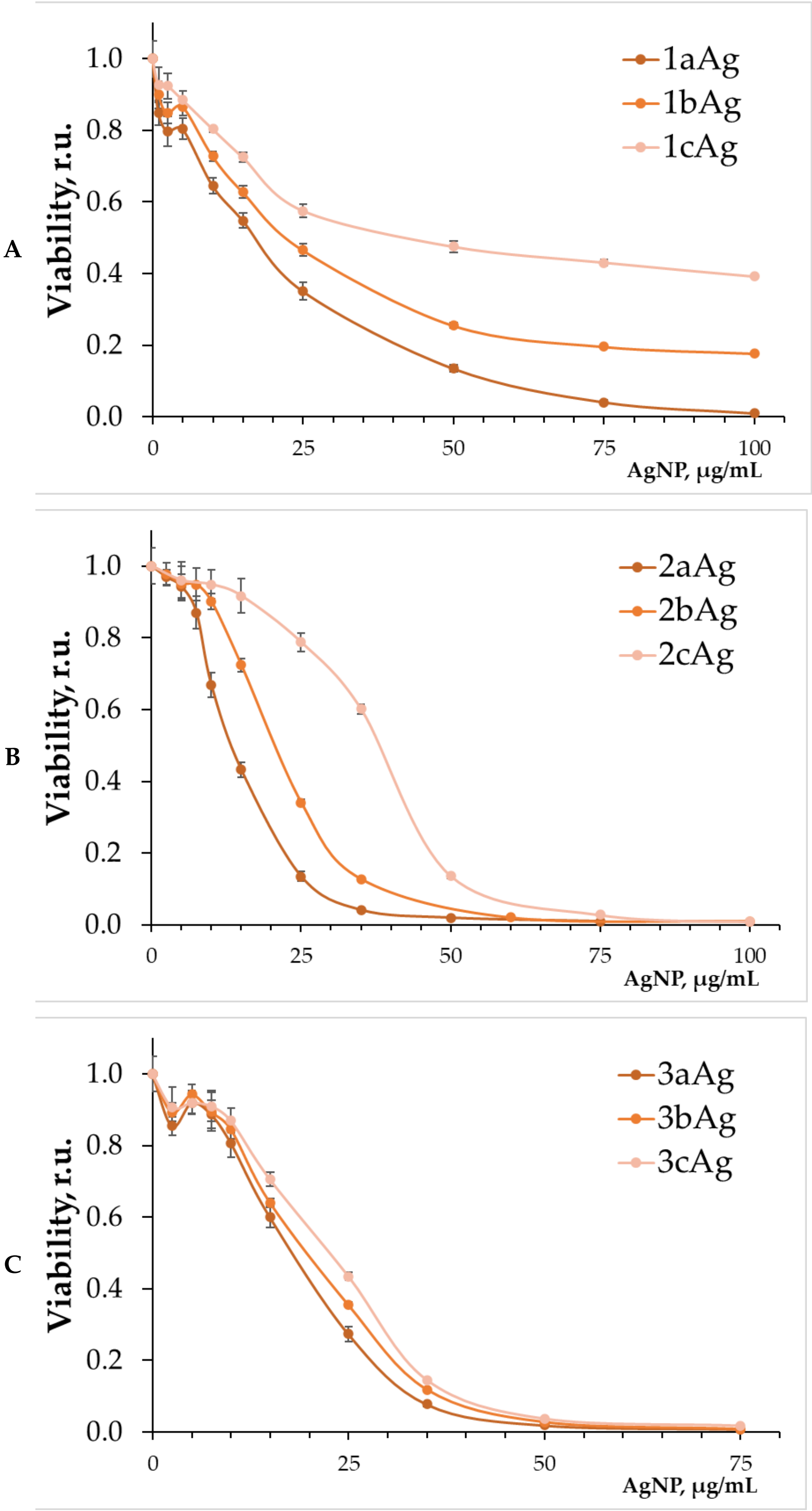

Figure S30. Dose-response curves of CEM-SS cells after 72 h incubation with AuNP *per se* with (A) 1st generation; (B) 2nd generation; (C) 3rd generation surface dendrons. Data obtained from MTT assay normalized to control (untreated) cells.

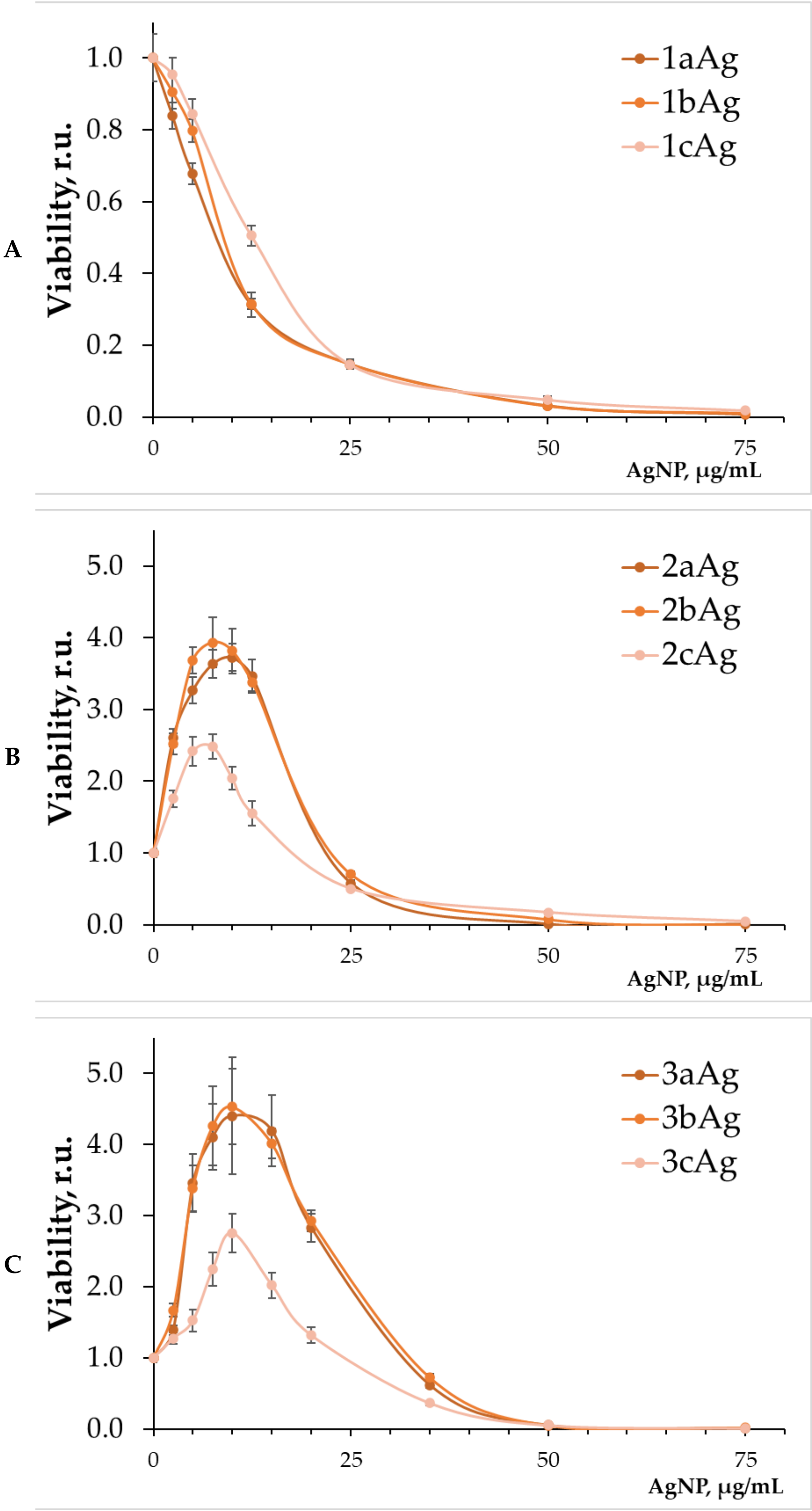

Figure S31. Dose-response curves of HeLa cells after 72 h incubation with AuNP *per se* with (A) 1st generation; (B) 2nd generation; (C) 3rd generation surface dendrons. Data obtained from MTT assay normalized to control (untreated) cells.

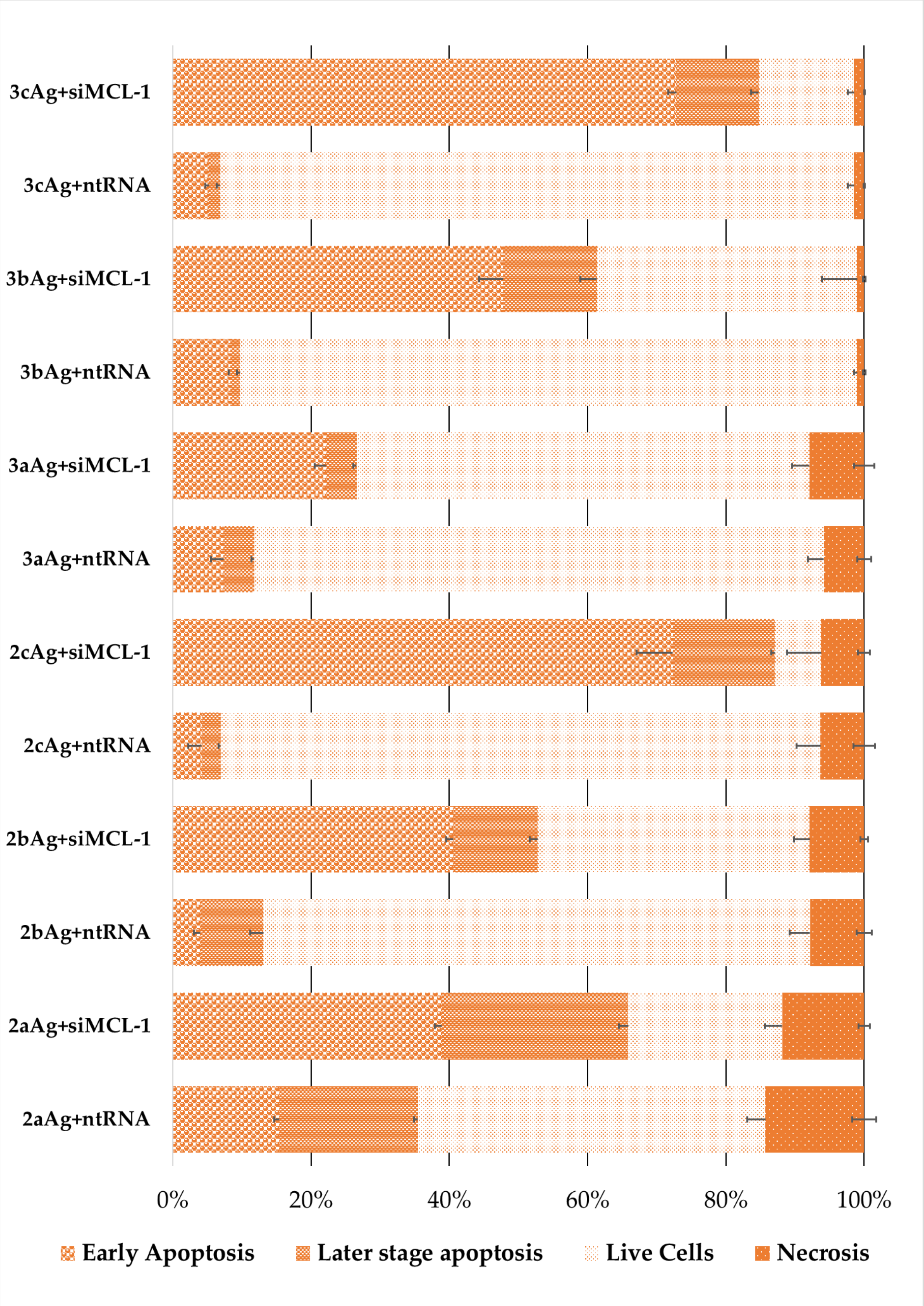

Figure S32. Distribution of HL60 cells by types of cell death among the FAM-positive cells after 48 h incubation. AgNP concentrations correspond to optimal delivery concentrations and equal to 25 μg/mL for AgNP-G2 and 15 μg/mL for AgNP-G3; siRNA concentration equal to 100 nM. Data obtained by flow cytometry. In each repeat 100,000 events were collected and analyzed.

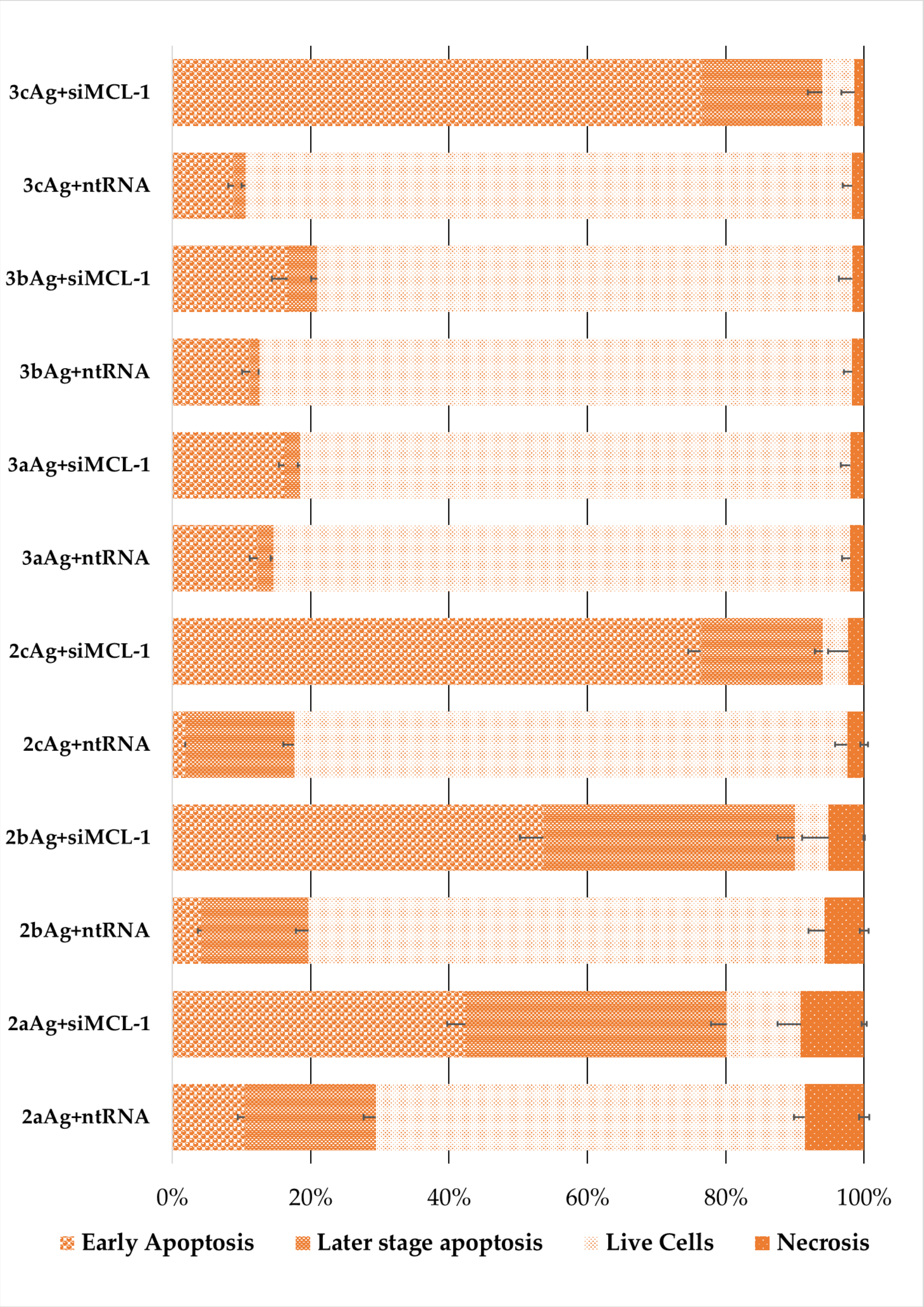

Figure S33. Distribution of CEM-SS cells by types of cell death among the FAM-positive cells after 48 h incubation. AgNP concentrations correspond to optimal delivery concentrations and equal to 25 μg/mL for AgNP-G2 and 15 μg/mL for AgNP-G3; siRNA concentration equal to 100 nM. Data obtained by flow cytometry. In each repeat 100,000 events were collected and analyzed.

A

B

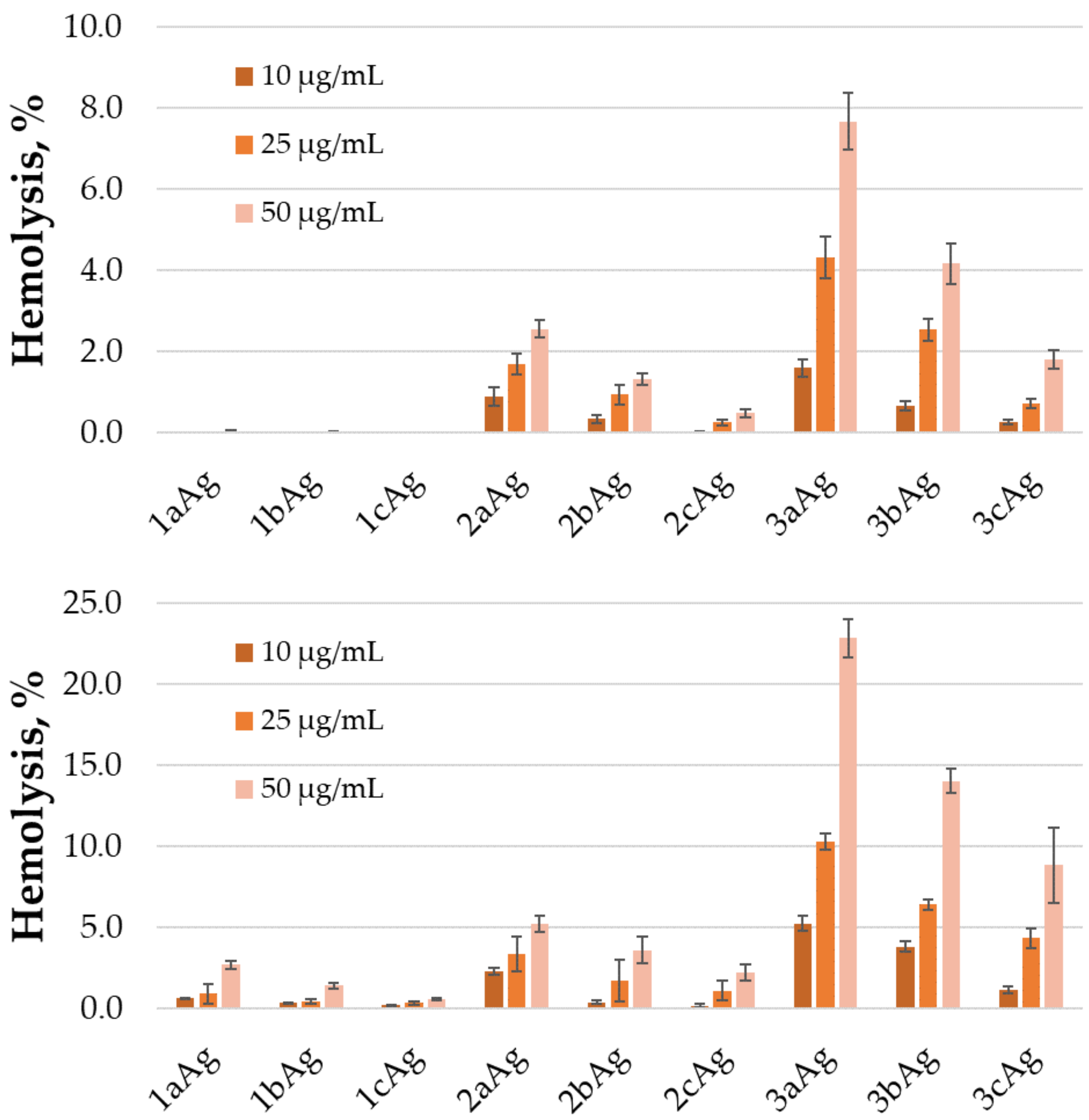

Figure S34. Hemolysis induced by AgNPs after (**A**) 2 h and (**B**) 24 h of incubation. Concentration of all AgNP equal to 50 μg/mL. Data presented as percentage of hemolysis, mean ± SD, n = 4.
